# Untangling the multi-regime molecular mechanism of verbenol-chemotype *Zingiber officinale* essential oil against *Aspergillus flavus* and aflatoxin B_1_

**DOI:** 10.1038/s41598-021-86253-8

**Published:** 2021-03-25

**Authors:** Prem Pratap Singh, Atul Kumar Jaiswal, Akshay Kumar, Vishal Gupta, Bhanu Prakash

**Affiliations:** 1 Centre of Advanced Study in Botany, Institute of Science, Banaras Hindu University, Varanasi, 221005 India; 2 Department of Biochemical Engineering and Biotechnology, Indian Institute of Technology Delhi, New Delhi, 110016 India

**Keywords:** Computational biology and bioinformatics, Microbiology

## Abstract

Aflatoxin B_1_ (AFB_1_), the natural polyketide produced by *Aspergillus flavus*, has a potent carcinogenic effect on humans as well as animals. In the present study, the antifungal and anti-aflatoxigenic B_1_ activity of chemically characterized *Zingiber officinale* essential oil (ZOEO) was investigated via in vitro analysis aided with molecular dynamics (MD) approaches. The GC–MS results revealed verbenol (52.41%) as the major component of oil. The antifungal and anti-aflatoxigenic activity of ZOEO was found to be 0.6 µl/ml and 0.5 µl/ml respectively. In-vitro analysis targeting the cell membrane, mitochondria and carbohydrate catabolism elucidated the probable antifungal mode of action. Further, docking and MD simulation results confirmed the inhibitory action of verbenol on the structural gene products (*Nor-1*, *Omt-1,* and *Vbs*) of aflatoxin biosynthetic machinery. Biochemical assays revealed the fungitoxic potential of the ZOEO while, computational results infers the stabilizing effects on the gene products upon verbenol binding leads to the impairment in its functionality. This is the first attempt to assess the multi-regime anti-AFB_1_ mechanism of verbenol chemotype-ZOEO targeting the *Nor-1*, *Omt-1,* and *Vbs* via computational approaches.

## Introduction

*Aspergillus flavus*, the predominant aflatoxin B_1_ producing species, is frequently present in the soil, agricultural crops and food products. With the ability to colonize the plant seeds, it poses the major safety problems for humankind and livestock. Being a food-borne fungus, it produces diverse secondary metabolites (aflatoxin B_1_ (AFB_1_) and cyclopiazonic acid) which contaminate and deteriorate feeding stuff^1^. Aflatoxin B_1_ is a potent mycotoxin that cause acute toxicity to the human lungs, kidneys, and colons of rodents^1^. According to the available literature, AFB_1_, among all the forms of aflatoxins, causes most severe health-related problems viz*.* acute intoxication, hepatotoxicity, teratogenicity, and immunosuppressive effects along with carcinogenicity^2^. The Joint FAO/WHO Expert Committee on Food Additives (JECFA) explained the carcinogenicity of aflatoxin in terms of ‘potency factor’ for cancer development, i.e., 0.01 cases/year per 100,000 people per ng AFB_1_ kg^−1^ body weight per day^3^. Because of all these traits, aflatoxins (specially AFB_1_) are set forth to be a major threat for humans as well as animals in the global context.

A range of physical and chemical management approaches have been practiced to reduce the risk factor associated with post-harvest aflatoxin contamination. However, all the strategies end up with several limitations like residual toxicity, microbial resistance, and loss of sensory and nutritional properties of food commodities^4^. In this context, the use of ‘green chemicals’ especially plant-based compounds would have better prospects as a safe and efficient control method of AFB_1_. Aromatic plant harbor essential oils (EOs: valuable secondary metabolites) that possess tremendous biological activity against harmful microbes such as pathogenic bacteria, molds, viruses, and pests^5^. Being natural in origin, some of the EOs and their constituent compounds viz*.* thymol, eugenol, estragole, anethole, and verbenol are considered environmentally acceptable, and listed as “Generally Recognized as Safe” (GRAS) by the Joint FAO/WHO Expert Committee on Food Additives (JEFCA) for mankind^6^.

*Zingiber officinale* Roscoe is one of the oldest medicinal herbs exhibited a wide range of bioactivity such as anti-cancerous, neuroprotective, antidiabetic, cardiovascular protective, and antiemetic^7^. Furthermore, the antifungal activity of *Zingiber officinale* essential oil (ZOEO) has already been reported against *Fusarium verticillioides, F. moniliforme, F. oxysporum, Candida albicans, Penicillium chrysogenum, Aspergillus solani, A. oryzae, A. niger, A. ochraceus*, and *A. flavus*^8^.

In the past few decades, the use of *in-silico* techno-advancements, molecular dynamics (MD) has become a potential tool in elucidating the behavioral patterns, strength, properties of the protein, and their interactive sessions with ligand molecules. MD simulation studies are well suited to explain the working mode of action (MOA) of the bioactive compounds in the context of drug designing^9^. In the molecular context of aflatoxin biosynthetic machinery, the products of structural genes such as *aflK* (AFLA_139190), *aflP* (AFLA_139210), and *aflD* (AFLA_139390), i.e.,* VERB* synthase (*Vbs*), *Omt-1* and *Nor-1* respectively, are the prime targets to elucidate the molecular MOA of bioactive compounds^10–12^. Therefore, investigation of ligand specificity and interactive signature receptor residues is essential to decipher the inhibitory MOA of the bioactive compounds (ligands).

**Figure 1:**
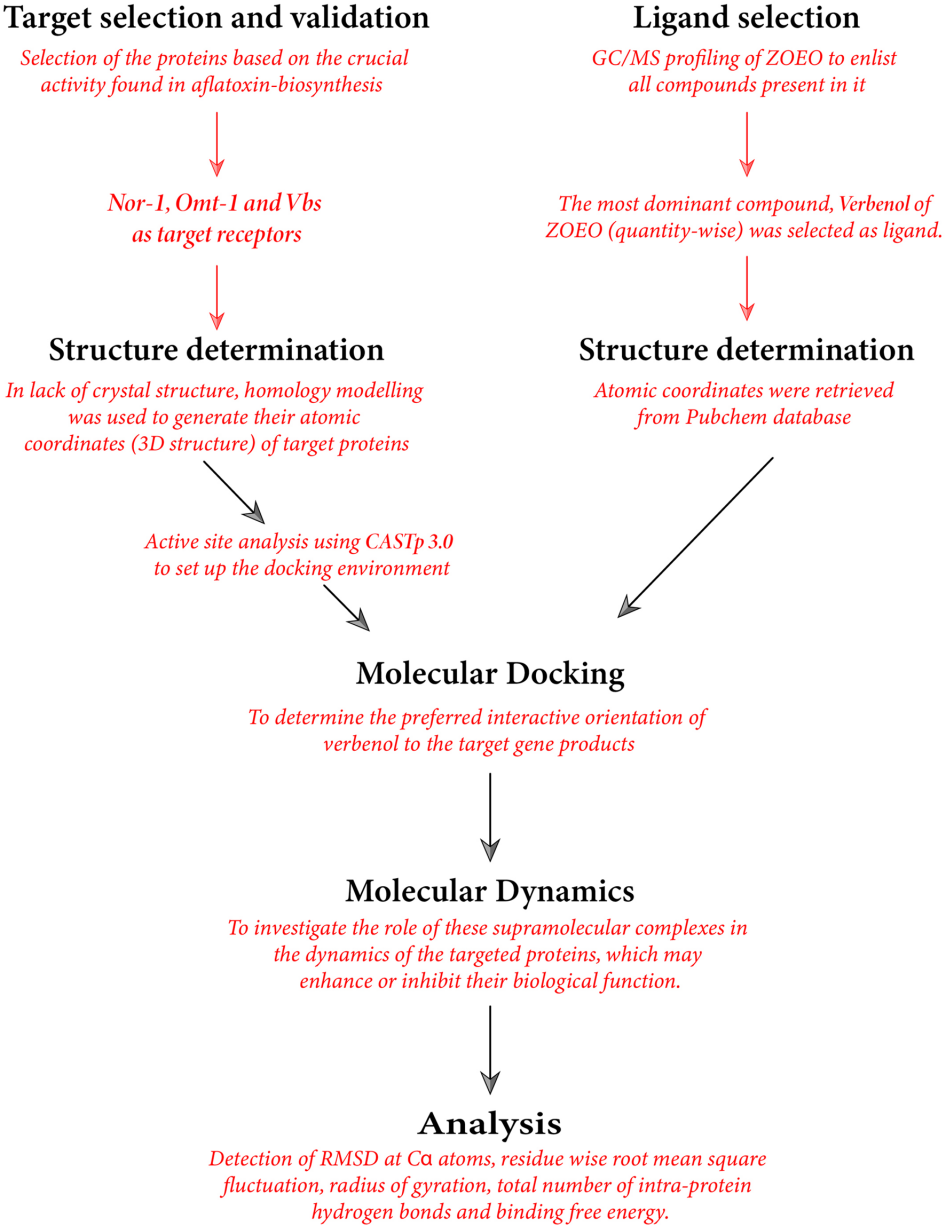
Methodological scheme summarizing the flow-chart of computational analysis.

The present study aimed to reveal the antifungal and anti-AFB_1_ potential of chemically characterized ZOEO by deciphering the mechanism underlying its fungicidal and aflatoxin B_1_ inhibitory actions. The fungal plasma membrane, mitochondria and cell carbohydrate catabolism were taken as the prime targets for the biochemical assays, with the intent of investigating the mechanism of antifungal activity of ZOEO. While, to understand the multi-regime anti-AFB_1_ action of ZOEO at molecular level, the three major gene products *Nor-1, Omt-1,* and *Vbs* that are critical in aflatoxin B_1_ biosynthetic cascade were targeted*.* Due to the lacking of the crystal structure of protein–ligand complex, docking protocols were simulated to predict the complex matrix defining the favored binding poses (energetically) of the selected ligand (compound) on the targeted receptor proteins (*Nor-1, Omt-1,* and *Vbs*). Furthermore, MD simulations were used to investigate the role of these supramolecular complexes in the dynamics of the targeted proteins, which may enhance or inhibit their biological function. The methodological scheme summarizing the computational studies in this paper has been presented in Fig. 1.

## Results

### Chemo-typing of essential oil and validating its antifungal and anti-aflatoxigenic activity

#### Chemical profile of ZOEO

**Figure 2:**
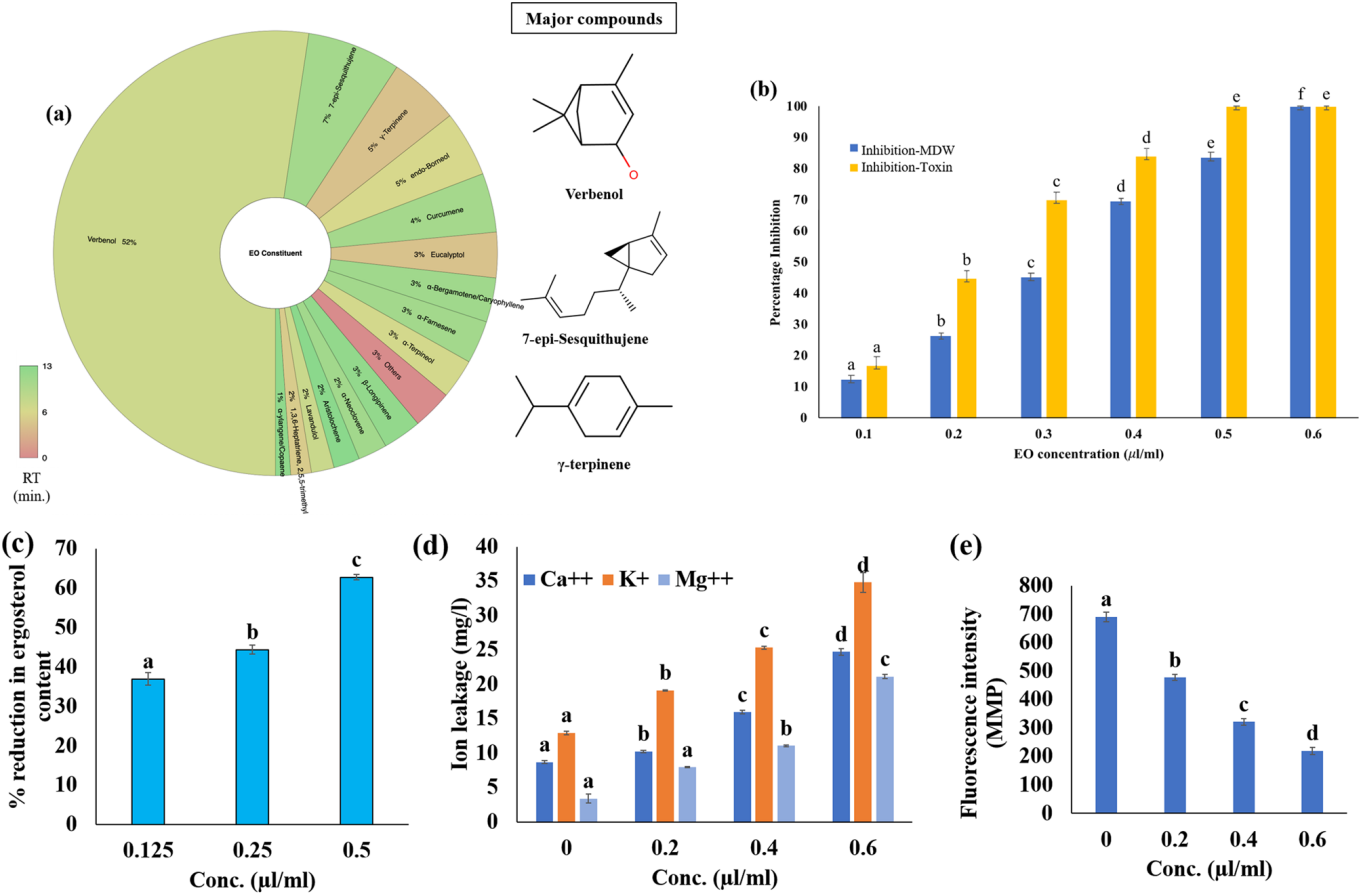
In-vitro analysis: (**a**) chemical profile of ZOEO; (**b**) anti-fungal and anti-aflatoxigenic activity of ZOEO; (**c**) % inhibition of ergosterol content; (**d**) vital cellular ion leakage; (**e**) mitochondrial membrane potential.

*Zingiber officinale* rhizome EO was isolated with the hydro-distillation process with yield of 12.32 ml/kg. GC–MS analysis revealed a total of 15 volatile components constituting ~ 99% of the complete chemical profile of the isolated ZOEO. The significant peaks were identified as the verbenol (52.41%) followed by 7-epi-Sesquithujene (6.8%), and γ-terpinene (5.18%) (Fig. 2a).

#### Antifungal and anti-aflatoxigenic activity: biochemical analysis

The antifungal activity of the ZOEO was calculated in terms of minimum inhibitory concentration (MIC) against the *A. flavus* PN-05. The effect of ZOEO on fungal dry weight and aflatoxin production was shown in Fig. 2b. The MIC of ZOEO was found to be 0.6 µl/ml; however, its inhibitory activity was recorded at 0.5 µl/ml for the AFB_1_ content (Fig. 2b). The decrease in mycelial dry weight and AFB_1_ content was in direct proportion with the doses of ZOEO.

The fungitoxic mechanism of ZOEO was elucidated through biochemical analysis of membrane integrity (perturbance in ergosterol content and membrane cations), mitochondrial membrane potential (MMP) and carbohydrate catabolism.

The efficacy of ZOEO in perturbing the cell membrane integrity of *A. flavus* was deciphered by its effect on production of ergosterol and perturbance in membrane cations (Ca^++^, K^+^ and Mg^++^) flow. The percent reduction of ergosterol content was found to be 37.95%, 44.38% and 62.77% respectively, exposed to 0.125, 0.25 and 0.5 µl/ml doses of ZOEO, compared with the control (Fig. 2c); while, the increase in the ion leakage was observed from the mycelia of the *A. flavus* fumigated with the ZOEO (Fig. 2d).

The MMP plays an important role in energy-coupling phenomena of mitochondria via generation of ATP. The reduction in the fluorescence intensity of the dye reflects the degradation of MMP. The reduction in the fluorescence intensity of the *A. flavus* cells exposed to different doses of ZOEO (0.2, 0.4 and 0.6 µl/m) can be easily visualized in Fig. 2e.

**Figure 3:**
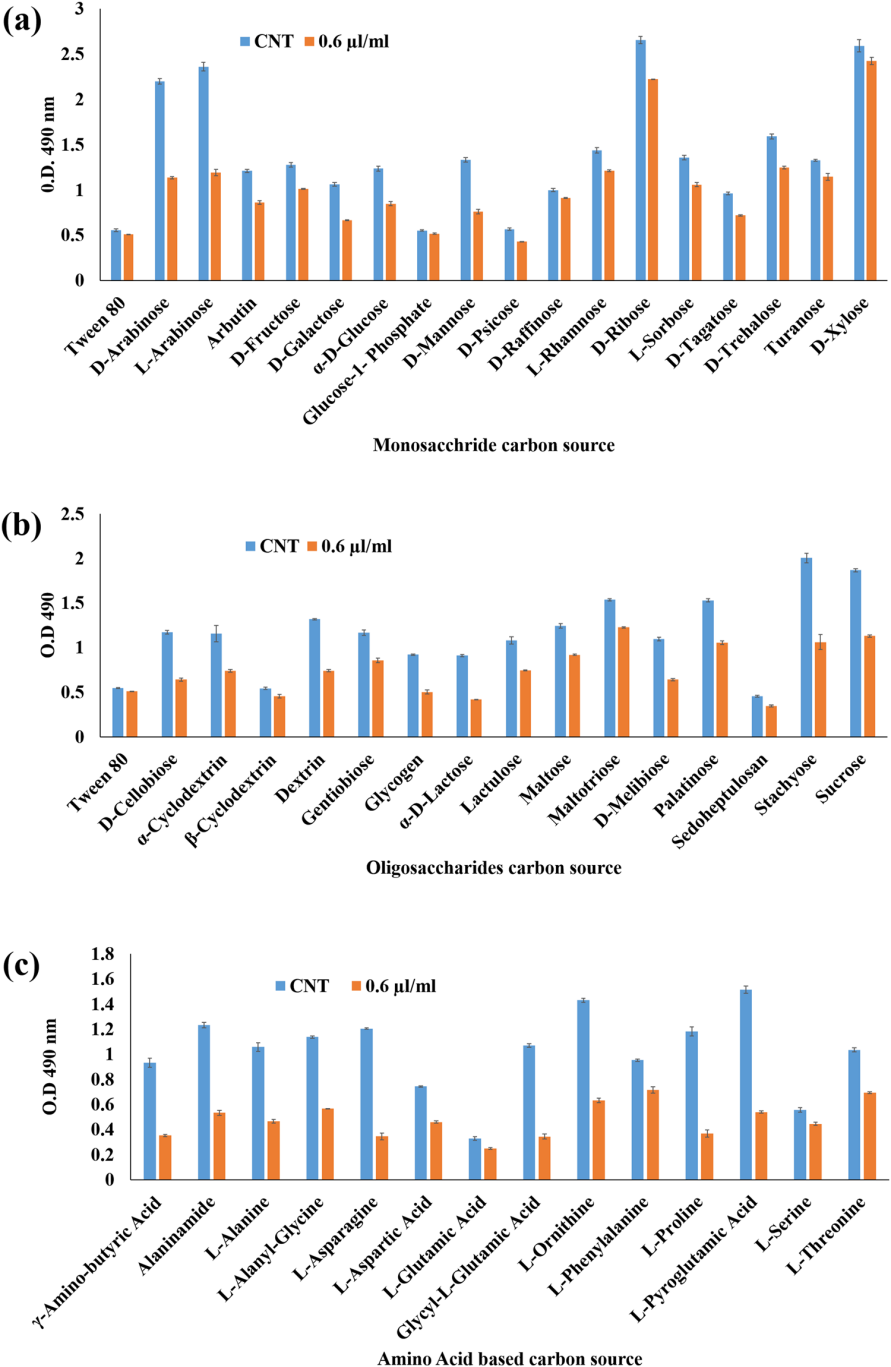
Carbon sources utilization pattern of *A. flavus* exposed to ZOEO (**a**) monosaccharide C-sources; (**b**) disaccharide C-sources; and (**c**) amino-acid based C-sources.

There were three categories of the carbon sources (monosaccharide, oligosaccharide and amino based carbon sources) which were the soul carbon sources of metabolic pathway. Therefore, these carbon sources were analyzed to visualize the alterations in the carbohydrate catabolism of the fungal cell exposed to ZOEO (0.6 µl/ml). The result indicated that the d-Arabinose, l-Arabinose, d-Ribose, d-Trehalose, d-Xylose (monosaccharide), Maltotriose, Palatinose, Stachyose, Sucrose, (oligosaccharide), and Alaninamide, l-Asparagine, l-Ornithine, l-Proline, l-Pyroglutamic acid (amino based) carbon sources of control sets were highly utilized over the other moderately or least utilized carbon sources. Whereas, the *A. flavus* cells treated with ZOEO showed the significant decline in the utilization of the above-mentioned carbon sources (Fig. 3).

### Molecular docking: the binding affinity of the receptor-ligand systems

#### Target proteins selection

The selection of the target proteins was based on their crucial activity reported in aflatoxin-biosynthesis in the previous studies with *A. parasiticus* and *A. flavus*^10–12^. Therefore, to decipher the preferred mechanism of aflatoxin biosynthetic inhibition, *Nor-1, Omt-1*, and *Vbs* were selected as the target proteins. They serve as the potential targets to assess the anti-aflatoxigenic activity of the selected compound (verbenol) as they perform the essential functions in aflatoxin biosynthesis^12–16^. The *Nor-1* (*aflD* gene product), a short-chain dehydrogenase/reductase enzyme is required to convert the norsolorinic acid (NOR) into the averantin (AVN)^13^. Papa^13,14^ demonstrated the conversion of NOR to AVN as one of the critical intermediate steps for the aflatoxin production, while Trail et al.^15^ proved the involvement of the *aflD* (*Nor-1*) in aflatoxin production. Another selected target protein in the pathway was *Vbs* (VERB synthase: *aflK* gene product). This enzyme catalyzes the synthesis of versicolorin B (VERB) from versiconal (VAL), another key step in aflatoxin biosynthesis^16^. *Vbs* was held responsible for aflatoxin’s toxicity and carcinogenicity^12^. The *Omt-1/Omt-A* (*aflP* gene product), a methyltransferase enzyme play a crucial role in the later steps of the biosynthetic pathway, catalyzes two reactions in the pathway, i.e., conversion of sterigmatocystin (ST) to o-methylsterigmatocystin (OMST) and dihydrosterigmatocystin (DHST) to dihydro-o-methylsterigmatocystin (DHOMST)^16,17^. These steps are the second last steps for the synthesis of aflatoxins (AFB_1_ from OMST and AFB_2_ from DHOMST). Significance of *Omt-1* can be seen with the fact that, *Aspergillus nidulans* has ST as the end product rather than aflatoxins since it lacks an *aflP* orthologue^12^.

#### Homology modeling and model validation

In the present study, the target proteins *Nor-1, Omt-1*, and *Vbs* were lacking the crystal structure, no hits were found when searched with the keywords ‘*Nor-1*’, ‘*Omt-1*’, and ‘*Vbs*’ converging the search on ‘*Aspergillus flavus*’ on the RCSB-PDB website. Therefore, homology modelling was used to generate the atomic coordinates (3D structure) of the target proteins to perform the further computational studies^18,19^. For homology modeling, templates were identified subjecting the maximum query (target protein FASTA sequence) coverage, maximum identity (PSI-BLAST), and maximum hits by pGEN THREADER as the filters. The selected templates were 3WXB, 6IX8, and 5NCC for *Nor-1*, *Omt-1*, and *Vbs,* respectively. At last, 3D models having stable and functional quality structures were filtered out based on the highest GMQE and lowest QMEAN scores (Fig. SM1)^20^.

The stereochemical-stability validation of the 3D models of *Nor-1, Omt-1*, and *Vbs* gave the overall quality score of 86.7, 88.3, and 89.07, respectively, out of 100 scoring function of SAVES v5.0. The Ramachandra plot statistics validate the acceptance of the 3D models (Fig. ST3). For reliability of the 3D models, their validity has been cross checked by analyzing their RMSD values in reference to their template crystal structures^21,22^. *Nor-1* superimposed with protein PDB ID: 3WXB having RMSD 0.586, *Omt-1* superimposed on protein PDB ID: 6IX8 with RMSD 0.713 and *Vbs* showed RMSD of 1.524 on superimposition with PDB ID: 5NCC (superimposed images: Fig. SM2). The RMSD less than 2 Å indicates a close homology and ensures reliability of the model in reference to the experimental data. ProTSAV^23^ assesses the quality of the protein structure at various interfaces viz*.* PROCHECK^24^, ProSA^25^, ERRAT^26^, Verify3D^27^, and ProQ^28^. It revealed the stable stereochemical properties of all the 3D models with RMSD values in the range of good model (green-yellow zone) (Fig. SM3). Further, protein information concerning its motifs, helices, strands, domains, tunnels, angles, positions, and errors were assessed via PDBsum^21^ web-tool to decipher the additional features of the target proteins (Fig. SM4).

#### Active site analysis

The active site analysis is a crucial task in view of setting the docking environment and ensuring the reliability of the docking analysis. However, due to lack of the crystal structure of receptor-ligand complex, computational algorithms were used to identify the active site of the proteins. In the present study, CASTp 3.0 tool, working on alpha-shape theory, was used to investigate the surface region details of a protein to analyze its interactive function with other molecules (ligand)^29,30^. CASTp 3.0 tool predicts the interior voids and surface pockets of proteins. These internal cavities of proteins are of great interest in discovery of small drug-like molecules that are associated with their binding events^31–33^. For all the target proteins, the binding pocket selection was made following the coordinated study of structure information obtained from the PDBsum server and CASTp 3.0 webtool. The pockets selected for *Nor-1, Omt-1*, and *Vbs* contain the maximum number of the functionally critical secondary structures (Fig. SM5).

#### Ligand selection and its toxicity estimation (ADMET properties)

Verbenol being > 50% of the total chemical profile of the ZOEO and all the other compounds had < 10% in quantity individually (Fig. 2a). Therefore, it can be stated that the isolated ZOEO was of verbenol-chemotype. Hence, verbenol was selected as the ligand for in-silico assessment of the working MOA of anti-aflatoxigenic activity of ZOEO.

The toxicity assessment of the compound is used to estimate its deleterious effects over both human health and the environment. Verbenol was assessed for determining its effects on human health, environment, and other ecological factors. Swiss Target Prediction^34^, Molinspirtation server, PREADMET, QikProp module (QikProp, Schrodinger LLC, NY, 2017), TEST (Toxicity Estimation Software Tool) of US Environmental Protection Agency and VEGA v1.1.5 were assessed to generate a comparative result analysis for more accurate result prediction. Based on the results shown in Table 1, verbenol does not seem to violate the measured parameters of safety (if provided at the acceptable range) in all the *in-silico* protocols used, it is also biodegradable, non-mutagenic, non-hepatotoxic, non-carcinogenic and non-tumorigenic. All the parameters have been calculated and verified for compliance with their standard ranges.

**Table 1:** ADMET profiling and ecological risk assessment.

**QikProp module, TEST, and VEGA v1.1.5 results**			
PubChem ID	61126	PlogS	− 2.384
Reactive functional groups	0	PlogHERG (IC_50_)	− 2.65
CNS	1	PCaco (nm/s)	4076.554
MW (g/mol)	154.252	PlogBB	0.19
Molecular formula	C_10_H_16_O	PMDCK (nm/s)	2259.46
SASA (Å^2^)	378.466	PlogKp	− 2.173
FOSA	337.803	#metab	1
FISA	40.663	PlogKhsa	− 0.064
Volume (Å^3^)	621.779	Human oral absorption	3 (high)
H-bond donor	1	PSA	20.497
H-bond acceptor	1.7	Number of nitrogen and oxygen atoms	1
Cohesive interaction index in solids	0.004492	Rule of five	0
Globularity Index	0.930885	Rule of three	0
Oral rat LD_50_ mg/kg (predicted)	2547.06	Tumorigenicity	Negative
Mutagenicity	Negative	Carcinogenicity	Negative
Drug-induced liver injury (Hepatotoxicity)	Negative	Biodegradation	Positive
Persistence (sediment)	nP (70 days)	Persistence (water)	nP (10 days)
Persistence (soil)	nP (5 days)	Air half-life	0.4162 h
**Molinspirtation server (bioactivity)**		**Swiss target prediction (Homo sapiens)**	
GPCR ligand	− 0.18	Target	Probability
Ion channel modulator	0.03	Androgen receptor	0.61
Kinase inhibitor	− 1.42	Sodium-dependent noradrenaline transporter	0.51
Nuclear receptor ligand	− 0.17	Sodium-dependent dopamine transporter	0.51
Protease inhibitor	− 0.54	Muscarinic acetylcholine receptor M1	0.49
Enzyme inhibitor	0.02	Muscarinic acetylcholine receptor M2	0.43
**PreADMET (ADMET)**		Muscarinic acetylcholine receptor M3	0.43
hERG inhibition	Low risk	Muscarinic acetylcholine receptor M4	0.43
Carcino mouse/rat	Negative	Muscarinic acetylcholine receptor M5	0.43
CYP 2C19 inhibition	Inhibitor	Cytochrome P450 19A1	0.43
CYP 2C9 inhibition	Inhibitor	Cholinesterase	0.39
CYP 2D6 inhibition	Non	Acetylcholinesterase	0.39
CYP 2D6 substrate	Non	Sodium-dependent serotonin transporter	0.39
CYP 3A4 inhibition	Inhibitor	Tyrosyl-DNA phosphodiesterase 1	0.38
CYP 3A4 substrate	Non	Estrogen receptor	0.38
Human intestinal absorption (%)	100	Estrogen receptor beta	0.38

*MW* molecular weight, *SASA* total solvent accessible surface area, *FOSA* hydrophobic component of the SASA, *FISA* hydrophilic component of the SASA, *PlogS* predicted aqueous solubility (log S), *PlogHERG* predicted IC_50_ value for blockage of HERG K^+^ channels, *PCaco* predicted apparent Caco-2 cell permeability (model for the gut-blood barrier), *PlogBB* predicted brain/blood partition coefficient, *PMDCK* predicted apparent MDCK cell permeability, *PlogKp* predicted skin permeability (log Kp), *PlogKhsa* prediction of binding to human serum albumin, *PSA* Van der Waals surface area of polar nitrogen and oxygen atoms, *Rule of five* number of violations of Lipinski's rule of five, *#metab* number of likely metabolic reactions, *Rule of three* number of violations of Jorgensen’s rule of three (PlogS >  − 5.7, PCaco > 22 nm/s, #Primary Metabolites < 7), *nP* non-persistant.

#### Molecular docking: assessing the molecular affinity of verbenol against target proteins

**Figure 4:**
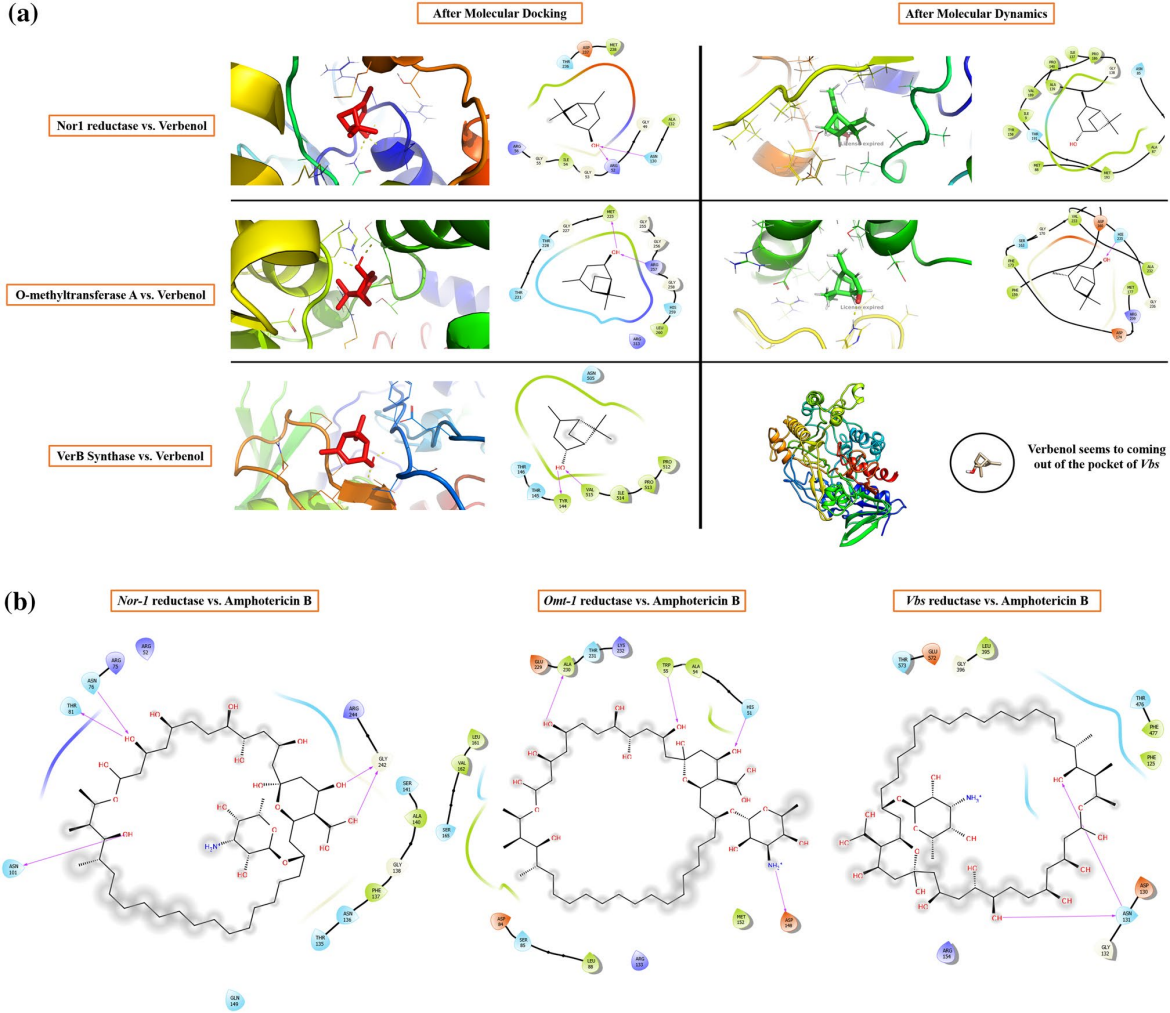

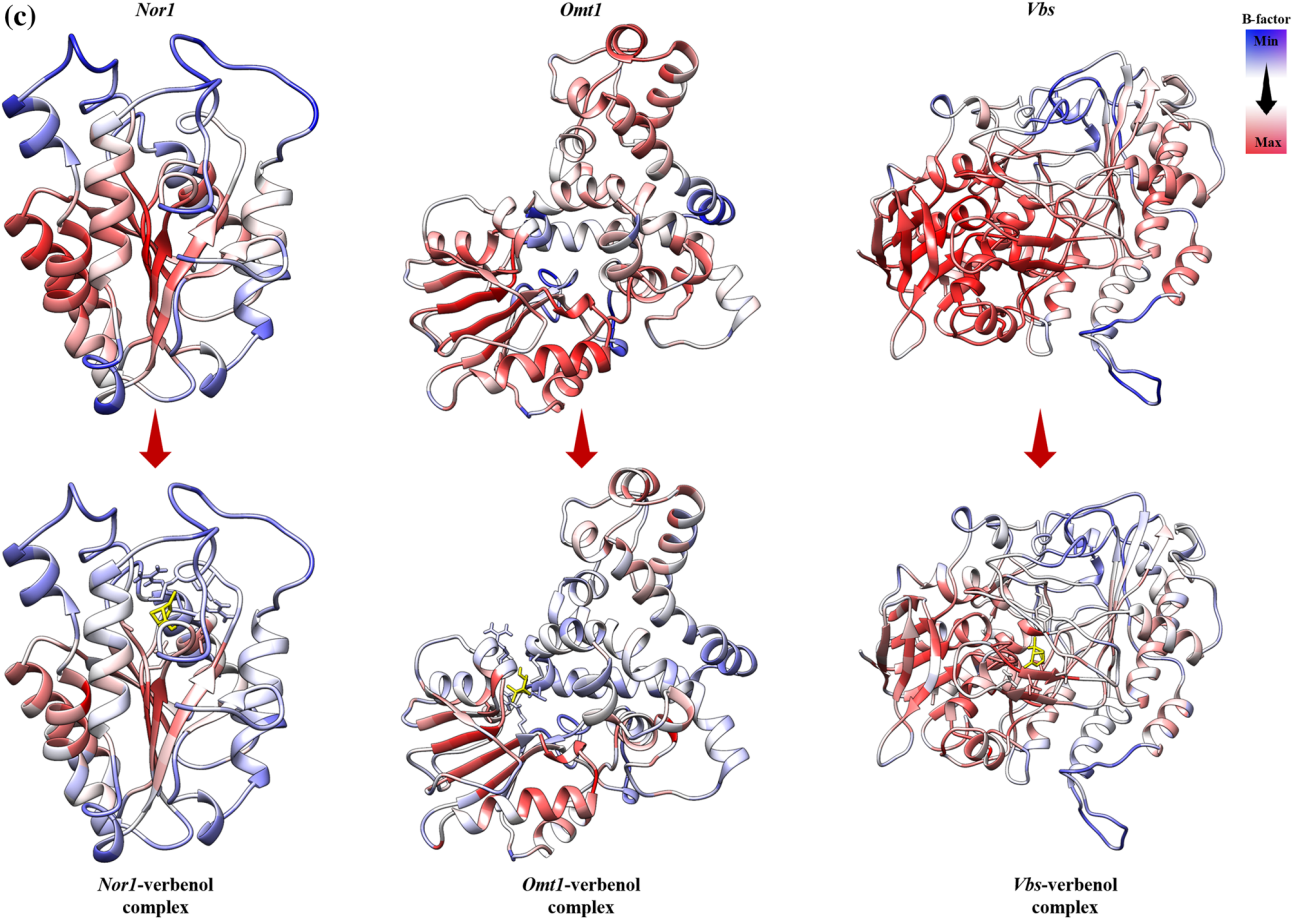
(**a**) Interactive behavior of target proteins with verbenol; (**b**) the binding details of amphotericin with target proteins; (**c**) B-factor profiling of proteins and protein-verbenol complexes.

The lack of crystal structures of receptor-ligand complexes for target proteins, led to use the docking algorithms to identify the binding position and the affinity persuaded between the small molecule (ligand) and the target proteins at the molecular level^35^. Prepared structure of the verbenol molecule was docked into the pre-adjusted docking environment in the target proteins as per the details given in Table 2. Binding predictions made by AutoDock were based on the empirical force field and the Lamarackian Genetic Algorithm, that scores the receptor-ligand binding positions in terms of the binding energy in kcal/mol (sum of the intermolecular energy and the torsional energy)^36^. The obtained docking energies and binding details were shown in Table 3, and binding poses were presented in Fig. 4a. Furthermore, for the validation of the predicted binding poses of the receptor-ligand complexes, all-atom MD simulation was performed^37^.

**Table 2:** Details of the docking environment used for the molecular docking study.

Receptor	Ligand*	Coordinates of the Grid Centre (Å)	Grid dimensions (Å)
*Nor-1*	Verbenol	X = 49.626	X = 104
		Y = − 10.685	Y = 86
		Z = − 15.266	Z = 106
	Amphotericin B	X = 47.426	X = 80
		Y = − 10.76	Y = 96
		Z = − 14.997	Z = 104
*Omt-1*	Verbenol	X = 23.525	X = 118
		Y = − 31.482	Y = 108
		Z = − 18.444	Z = 126
	Amphotericin B	X = 22.871	X = 116
		Y = − 31.482	Y = 108
		Z = − 19.501	Z = 126
*Vbs*	Verbenol	X = 34.635	X = 104
		Y = 38.764	Y = 82
		Z = 48.296	Z = 126
	Amphotericin B	X = 32.894	X = 122
		Y = 38.896	Y = 76
		Z = 48.664	Z = 124

**Table 3:** Docking details of all the three protein–ligand systems.

Interactions	Bond type	Resides and their legends	Binding energy (kcal/mol)	Inhibition constant (µM)	Ligand efficiency
Verbenol with *Nor-1*	Hydrogen bond	ARG52, GLY49, GLY55, ASN130	− 6.10	33.52	− 0.55
	Hydrophobic bond	MET238, ALA132, ILE54			
	Polar bond	ASN130, THR236			
	Charged bond	ASP237, ARG56, ARG52			
Verbenol with *Omt-1*	Hydrogen bond	MET225, ARG257, HIS259	− 5.81	55.49	− 0.53
	Hydrophobic bond	MET225, LEU260, GLY227, GLY255			
	Polar bond	THR228, THR231, HIS259			
	Charged bond	ARG257, ARG313			
Verbenol with *Vbs*	Hydrogen bond	TYR144, VAL515	− 5.56	84.62	− 0.51
	Hydrophobic bond	THR146, PRO512, PRO513, ILE514, VAL515			
	Polar bond	THR145, THR146, ASN505			
Amphotericin B with *Nor-1*	Hydrogen bond	ASN76, THR81, ASN101, GLY242	− 6.76	11.00	− 0.10
	Hydrophobic bond	PHE137, GLY138, ALA140			
	Polar bond	THR135, ASN136, SER141, GLN149			
	Charged bond	ARG52, ARG75, ARG244			
Amphotericin B with *Omt-1*	Hydrogen bond	HIS51, TRP55, ASP148, ALA230,	− 7.49	3.23	− 0.12
	Hydrophobic bond	ALA54, LEU88, MET152, LEU161, VAL162			
	Polar bond	SER85, SER165, THR231			
	Charged bond	ASP84, GLU229, LYS232			
Amphotericin B with *Vbs*	Hydrogen bond	ASN131	− 6.72	11.95	− 0.10
	Hydrophobic bond	PHE125, GLY132, LEU395, GLY396, PHE477			
	Polar bond	THR476, THR573			
	Charged bond	ASP130, ARG154

Verbenol showed the dynamic multi-regime inhibitory activity against the target proteins. It showed the highest binding affinity with the *Nor-1* followed by *Omt-1* and *Vbs*. Verbenol formed the hydrogen bonds with ARG52, GLY49, GLY55, and ASN130 in *Nor-1*, MET225, ARG257, and HIS259 in *Omt-1* and with TYR144, and VAL515 in *Vbs*. Based on the binding energy profile of verbenol, its Ki (inhibition constant) value for each target proteins were calculated following the Eq. (1).

1 $${\text{K}}_{{\text{i}}} = {\text{exp }}\left( {\Delta {\text{GR}}^{ - 1} {\text{T}}^{ - 1} } \right),$$

where ∆G = Binding energy in cal/mol, R = Gas Constant (1.9187 cal/mol/K), T = Temperature (300 K).

The Ki value is a valid parameter to measure the efficacy of the inhibitor towards the enzyme quantitively. Low Ki denotes a high binding affinity of the inhibitor^38^. Verbenol showed the lowest possible inhibition constant towards the *Nor-1,* followed by *Omt-1* and *Vbs* (Table 3). Furthermore, Ki values of verbenol against all the target proteins/enzymes only differed by a little amount, which represents the multi-regime inhibitory potential of the verbenol against aflatoxin biosynthesis.

#### Comparative analysis with Amphotericin B (commercial antifungal drug)

The commercial antifungal drug amphotericin B has been selected for the comparative study of molecular docking results. MIC of amphotericin B (1.8 µl/ml)^39^ was comparatively higher than the ZOEO (0.6 µl/ml) (Fig. 2a) A comparative study of binding energy, inhibition constant and ligand efficiency has been shown in Table 3. The results revealed that the amphotericin B showed the better efficacy in inhibiting the target proteins (*Nor-1*, *Omt-1*, and *Vbs*) that have crucial roles in AFB_1_ biosynthesis than verbenol. As well as, verbenol also showed remarkable binding affinity near to amphotericin B with target proteins which reveals its utility as a plant-based antifungal agent. The binding details of amphotericin with target proteins were represented in Fig. 4b. Since amphotericin B is commercially available and established antifungal drug, hence, we have not performed the molecular simulation analysis with amphotericin B.

### MD simulation approach: validatory investigation of receptor-ligand complexes

To study the effect of verbenol binding on the targeted gene products (i.e., *Nor-1*, *Omt-1* and *Vbs*) and establish their validity, all-atom molecular dynamics simulation of 300 ns for receptor-ligand complexes of target proteins was in reference to the modelled structures of the target proteins employed (except *Nor-1* for which 500 ns simulation length was used). A total of 2 µs simulation was achieved for all six systems under study. On the obtained trajectory, several calculations (structural, dynamical, and thermodynamic) were performed to understand the inhibitory potential of verbenol against *Nor-1*, *Omt-1,* and *Vbs*. In the following sections, we have shown that the effect of verbenol binding on *Nor-1*, *Omt-1,* and *Vbs* at structural, dynamical, and thermodynamics level.

#### Structural and dynamic behavior analysis

**Figure 5:**
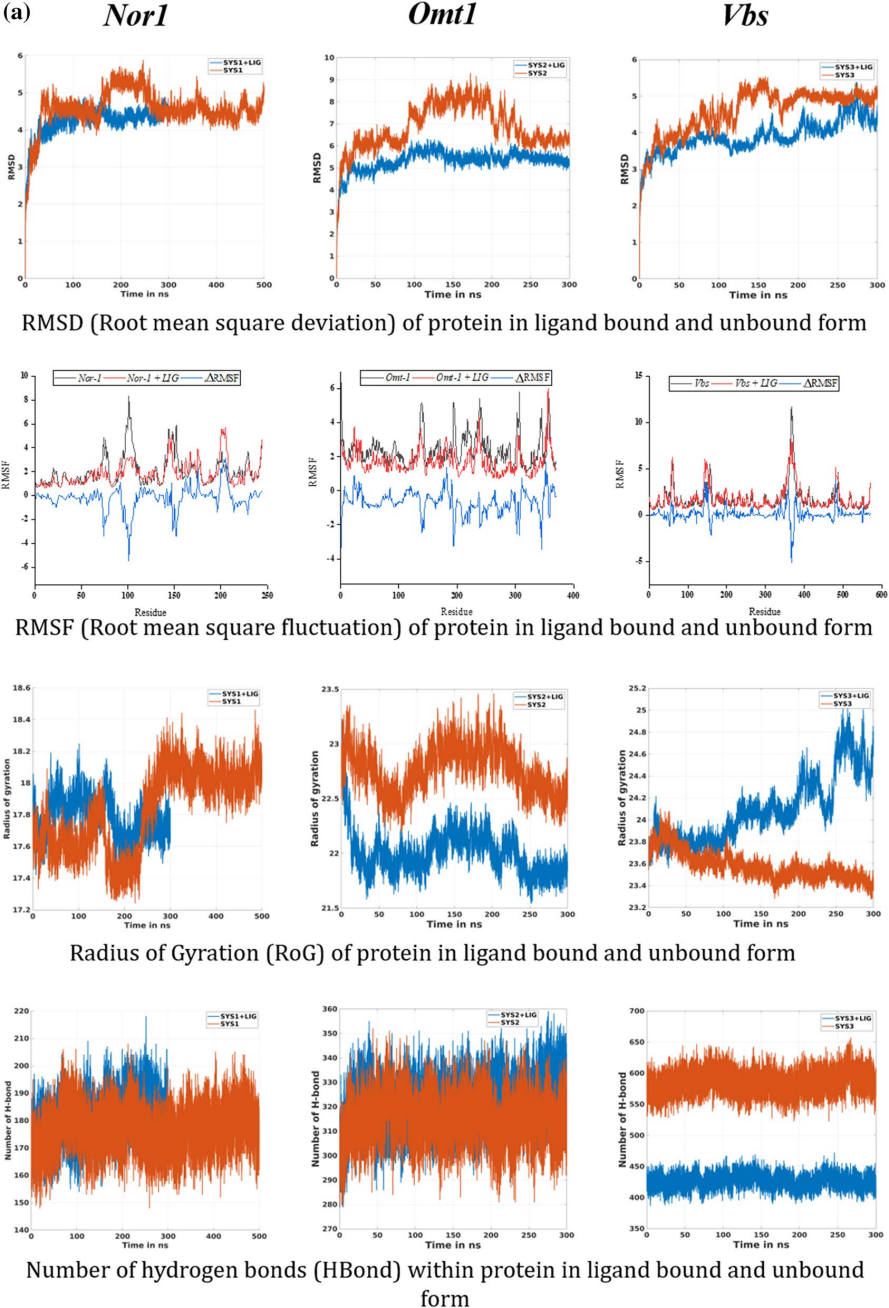

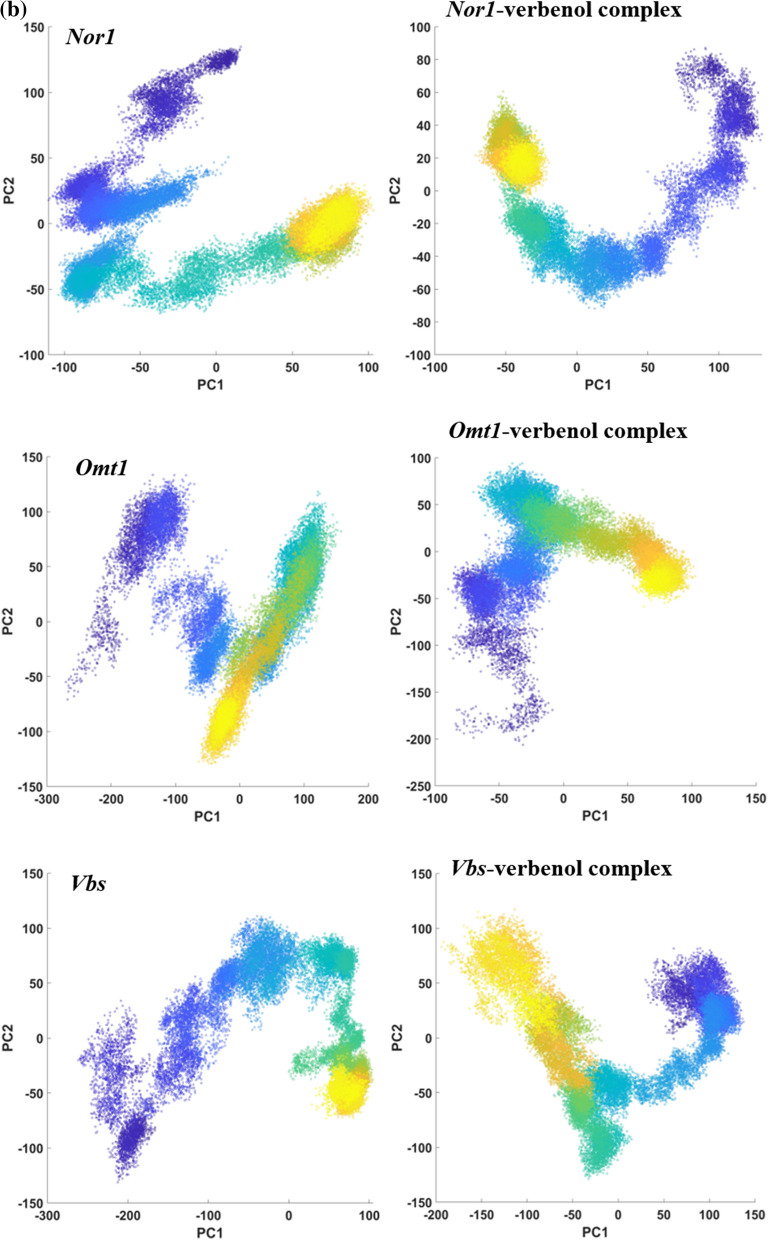

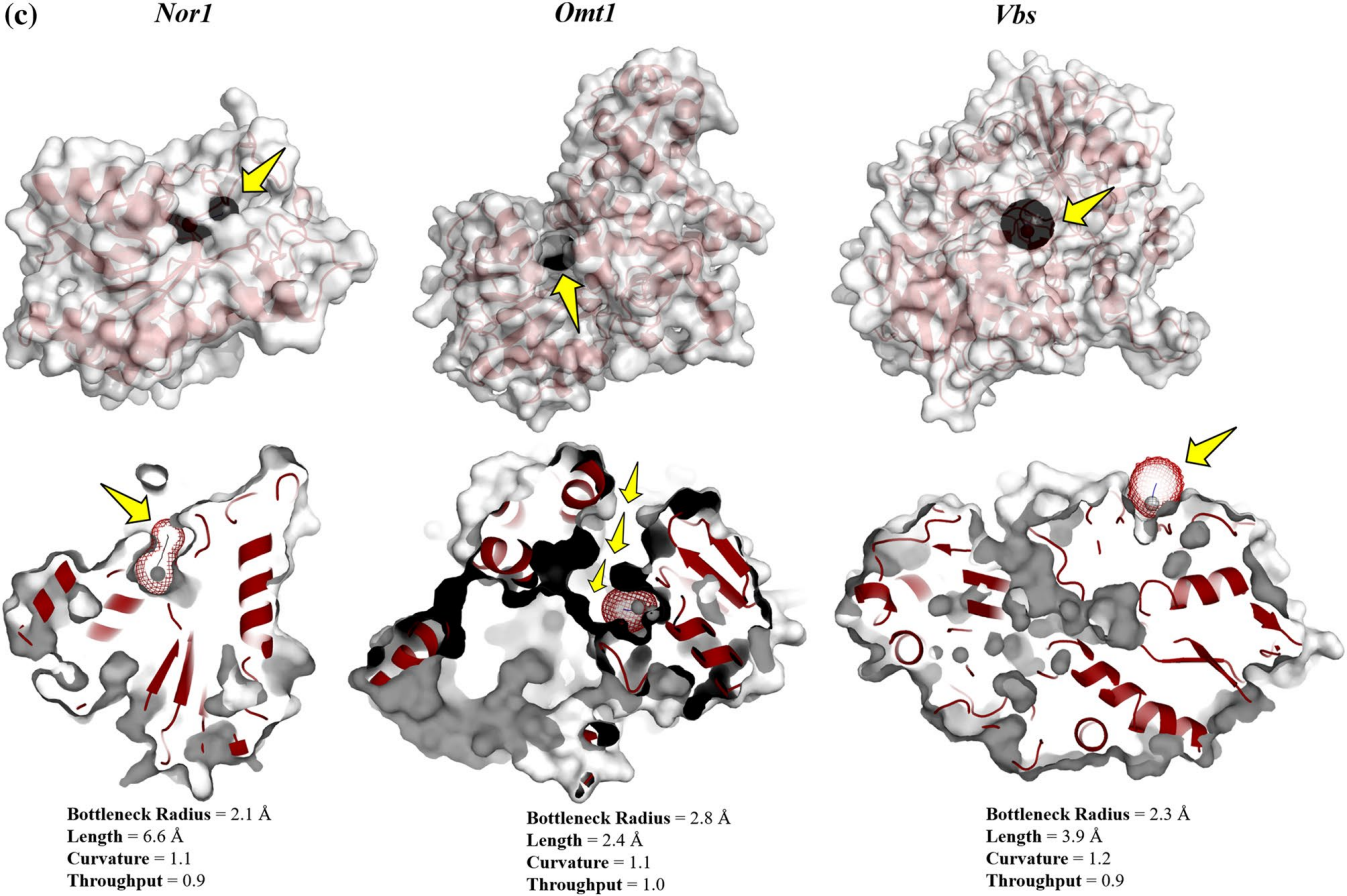
(**a**) Molecular dynamics simulation analysis; (**b**) PCA analysis; (**c**) tunnel analysis of protein–ligand complexes.

The structural behavior of the target proteins in their ligand-bound and unbound forms were assessed in the form of their backbone RMSD, Cα RMSF, B-factor, and Rg analysis. RMSD value quantifies the flexibility difference between two structures, considering one of them as reference structure (initial structural conformation was taken reference here). In terms of RMSD, protein structures having less deviation are the more stable ones, and vice versa^40,41^. As shown in Fig. 5a, all the three receptor-ligand systems attained the stability earlier than their reference proteins (without ligand) (~ 100 ns for both *Nor-1*/verbenol and *Omt-1*/verbenol, ~ 200 ns for *Vbs*/verbenol and ~ 250 ns for all the three target proteins *Nor-1, Omt-1*, and *Vbs*). With the inspection of the average RMSD values, complex systems showed stable conformations than their respective target proteins, which emphasizes the attainment of stability by target proteins on ligand binding. *Nor-1*/verbenol system showed lower RMSD (4.21 Å) than the *Nor-1* (4.57 Å), similarly, RMSD reported for the *Omt-1*/verbenol (5.31 Å), and *Vbs*/verbenol systems (3.89 Å) were lower than that of native target proteins *Omt-1* (6.84 Å) and *Vbs* (4.58 Å).

Likewise, the fluctuation of residues from their time-averaged position during the simulation was observed as their RMSF (root mean square fluctuation). It determines the flexibility of the proteins as a function of their residue numbers^40^. With the graphs presented in Fig. 5a, it could be seen that the RMSF values of the target proteins (*Nor-1* and *Omt-1*) were relatively higher in their ligand-unbound forms on the contrary to the ligand-bounded systems. In the case of the *Vbs*, the fluctuation seems to be neutral, emphasizing no effects of the ligand binding on the residue positioning. This was further investigated by the visual inspection of the trajectories, and the fluctuation of target proteins from their ligand-unbound form to ligand-bound form was presented as the ΔRMSF in the Fig. 5a.

The B-factor or Debye–Weller factor value of protein structure reflects its local motion, i.e., fluctuation from their average positions due to the kinetic energy of atoms^42^. The average B-factor fluctuation was calculated using the coordinates of the atoms (r) of the protein backbone from the Eq. (2).

2 $${\text{B}} = \left[ {8{\uppi }^{2} \left( {{\Delta r}^{2} } \right)} \right]/3.$$

The results showed in Fig. 4c, it could be inferences that the B-factor of the ligand-bound proteins were relatively lower than that of their ligand-unbound forms, i.e., the local motions of the atoms of the proteins were stabilizing upon ligand binding.

The radius of gyration (Rg) is the measure of protein compactness that works in an inversely proportional manner like RMSD^40^. The compactness of the protein differs in its bound and unbound states with the ligand. The Rg analysis of protein in ligand unbound and bound systems also support the previous finding (RMSF). The target proteins in their ligand unbound form portrayed the gyradius of 17.82 nm (*Nor-1*), 22.76 nm (*Omt-1*), and 23.57 nm (*Vbs*), while the proteins in ligand-bound form have the gyradius of 17.78 nm (*Nor-1*/verbenol), 22.0 nm (*Omt-1*/verbenol), and 24.10 nm (*Vbs*/verbenol). As it also can be seen in the Fig. 5a, Gyradius of the target proteins *Nor-1* and *Omt-1*, in their ligand unbound systems, were relatively higher than their ligand-bound form that characterizes the overall decrease in the compactness of protein–ligand binding. However, for the *Vbs*, there were higher gyradius for the ligand-bound form instead of ligand-unbound form.

Ligand binding might lead to some conformations changes in protein structure, to study the same we performed PCA analysis on obtained trajectories. With the help of PCA, we can characterize significant motions taking place in a protein. The eigenvector with the highest eigenvalue (PC1) captures maximum variance (largest collective motion) in the protein structure. In contrast, the eigenvector with the second largest eigenvalue (PC1) describes the second-largest collective motion in the protein. When we plot PC1 vs. PC2 (in 2D) obtain from MD simulation data, we observe similar conformations of protein falling into the same cluster. As can be seen from Fig. 5b, all the target proteins with or without ligand reach a stable conformation represented by the yellow cluster. However, the cluster form throughout 300 ns varies significantly with verbenol bound and unbound form. It showed that the conformational sampling of target proteins *Nor-1*, *Omt-1,* and *Vbs* varies significantly in verbenol bound and unbound form, which supports the study via RMDS, RMSF, Rg, and H-bond analysis.

#### H-bond analysis: the dynamic behavior of proteins

We assessed the total H-bonds of target proteins in the ligand bound and unbound states along with the ‘unique’ H-bonds between the target proteins and verbenol with 3 Å of donor–acceptor distance and 45° of angle cutoff which can significantly affect the protein stability^40^. The average number of hydrogen bonds detected in each frame as a function of time were 176 (*Nor-1*), 315 (*Omt-1*), 585 (*Vbs*) for ligand unbound states of proteins and 181 (*Nor-1*/verbenol), 322 (*Omt-1*/verbenol), and 426 (*Vbs*/verbenol) for their ligand-bound states. The H-bonding pattern deciphered the high number of H-bonding in the ligand-bound form of the proteins *Nor-1* and *Omt-1*. However, *Vbs* showed the higher H-bonds in its ligand unbound form, which lined in the result patterns of RMSF and gyradius analysis. The number of H-bonds formed in-between the verbenol, and target proteins were 31, 37, and 120 for the *Nor-1*/verbenol, *Omt-1*/verbenol and *Vbs*/verbenol systems, respectively. The details regarding these unique H-bonds were given in Supplementary Table ST1. There was only a single -OH group present in the structure of verbenol, which was involved in all the H-bonding with the target proteins. Therefore, there was a smaller number of H-bonds between the ligand and receptor proteins with minimal occupancy. Instead, *Nor-1*/verbenol and *Omt-1*/verbenol system showed the occupancy of some H-bonds above 10% while *Vbs*/verbenol system has all the H-bonds with occupancy < 3%.

In order to ensure the positioning of verbenol in the binding cavity of the target proteins in due course of simulation time, the distance between the center of mass (COM) of the ligand with the COM of the binding pocket of the target proteins as a function of time were assessed (Fig. SM6). Results assured the closeness of the COM of verbenol to the COM of binding pockets of the *Nor-1* and *Omt-1* proteins with an average distance of 13.23 Å and 17.7 Å while verbenol seems to be coming out of the *Vbs* binding pocket with an average distance of 34.65 Å.

#### Binding free energy analysis: binding selectivity prediction by MM/PBSA

In the enzyme-inhibitor systems, the efficacy of inhibitors to inhibit the enzymes are substantially related to the stability of their binding against enzyme. Therefore, the MD simulation study of the inhibitors should be evaluated in terms of their free energy of binding at the active sites of the target proteins. The binding affinity profiles of verbenol against target proteins were used for the selectivity of the best inhibitory action^43^. In the present study, the MD simulations were combined with the MM/PBSA approach to predict the binding free energies for all three receptor-ligand complex systems, i.e., *Nor-1/verbenol*, *Omt-1/verbenol*, and *Vbs/verbenol*. The combinatorial approach of MM/PBSA and MD simulations are thought to be more accurate to identify the correct binding conformations. With the results given in Table 4, the predicted binding free energies (Δ*G*_bind_) with Poison Boltzmann (PB) model for *Nor-1*/verbenol, *Omt-1*/verbenol, and *Vbs*/verbenol systems were − 2.95 ± 2.96, − 3.79 ± 4.05, and − 0.83 ± 3.17, respectively. These results impact the superiority of the *Nor-1*/verbenol and *Omt-1*/verbenol systems over the *Vbs*/verbenol. Along with, ΔE_EEL_ (change in electrostatic energy), ΔE_VDW_ (change in van der Walls energy), ΔG_PBSA_ (change in solvation energy, polar and non-polar contributions), and ΔG_bind_ (MM-PBSA binding free energy) also support the pattern of Δ*G*_bind_.

**Table 4:** The energy components predicted by MM/PBSA analysis (kcal mol^−1^).

	*Nor-1*	*Omt-1*	*Vbs*
ΔE_VDW_	− 22.73 ± 1.78	− 21.10 ± 2.59	− 11.73 ± 7.20
ΔE_EEL_	− 3.73 ± 2.83	− 5.25 ± 5.35	− 1.83 ± 3.78
ΔG_PBSA_	23.52 ± 3.34	22.56 ± 4.25	12.73 ± 7.67
ΔG_bind_	− 2.95 ± 2.96	− 3.79 ± 4.05	− 0.83 ± 3.17

*Mean ± SD.

*ΔE*_*EEL*_ change in electrostatic energy, *ΔE*_*VDW*_ change in van der Walls energy, *ΔG*_*PBSA*_ change in solvation energy both polar and nonpolar, and *ΔG*_*bind*_ MM-PBSA binding free energy.

#### Ligand pathway: CAVERDOCK analysis

The analysis of the pathway for the ligand (inhibitor) transport to the binding site of the protein holds a cornerstone in unlocking the conformational cascades taking place during the process. Furthermore, protein tunnels and channels are the key factors that assist the ligand passage to proteins’ external and internal environments. CaverDock, a fast-computational tool that facilitates the analysis of tunnel detection and ligand transport within a single environment, has been used in this study^44^. The ‘easiness’ in the ligand placement has been depicted in the form of energy values: E_Max_ (the highest binding energy in the trajectory) and E_a_ (activation energy of association: E_Max_ − E_Surface_ for reactants). The lower the E_Max_ and E_a_ values, the more the easiness in the ligand placement. The possible routes detected for the target proteins were shown in Fig. 5c, along with their energy profiles (Table 5).

**Table 5:** CaverDock energy profile of all three target proteins.

Parameters	*Nor-1*	*Omt-1*	*Vbs*
Bottleneck radius (Å)	2.1	2.8	2.3
Length (Å)	6.6	2.4	3.9
Curvature	1.1	1.1	1.2
Throughput	0.9	1	0.9
E_bound_ (kcal/mol)	− 5.2	− 4.7	− 3.7
E_max_ (kcal/mol)	0.4	− 4.7	− 3.3
E_surface_ (kcal/mol)	− 0.1	− 4.7	− 3.3
E_a_ (kcal/mol)	0.5	0	0
ΔE_BS_ (kcal/mol)	− 5.1	0	− 0.4

## Discussion

Prior to detailed investigation, ZOEO was chemically characterized by GC–MS, which revealed verbenol (52.41%) as the major compound of oil (Fig. 2a). The chemical profile of ZOEO was in accordance with the previous reports^45,46^. However, the quantity of the chemical constituents varied because of the various abiotic and biotic factors such as method of extraction, edaphic factors, age and part of plants^47^. Antifungal efficacy of ZOEO and verbenol has been previously reported by Prakash et al.^48^ and Al-Ja’Fari et al.^49^. However, detailed investigation on the molecular details of the AFB_1_ inhibition and mechanism of fungitoxic potential of ZOEO is still lacking in literature so far. Therefore, in the present study, the mechanism of fungitoxic potential along with the molecular mechanism of aflatoxin B_1_ inhibition/dysfunction of ZOEO was investigated via the biochemical and computational based approaches.

The present study showed the fungi-toxic nature of the ZOEO and its components (mainly verbenol) due to their negative implications on the fungal cell membrane, depolarization of the mitochondrial membrane and hampering of the carbohydrate catabolism pathway of the cell. The lipophilic nature of the EO made its entry through cell membrane resulting the damaging effects on the membrane integrity. The reduction in the ergosterol production in treated *A. flavus* cells (Fig. 2c), ensures the effect of the ZOEO on the major sterol of fungal cell membrane. OuYang et al.^50^ have previously reported that the EO or its component cause the downregulation of the significant genes involved in ergosterol biosynthesis. These phenomena might be responsible for the alteration in the permeability of the membrane and causing the leakage of the various cations as confirmed in the present study (Fig. 2d). The study also revealed the significant alteration in the membrane potential of mitochondria in a dose-dependent manner (Fig. 2e), which suggested the disruption in the functioning of mitochondria. The alterations in the MMP were observed using a cell permeable cationic dye, Rh123, which can penetrate into the mitochondria and reflect the MMP^51,52^. Above all, ZOEO may shattered the various metabolic pathways viz pentose phosphate pathway (PPP), glycolysis and tricarboxylic cycle (TCA) by declining the utilization of the significant carbon sources (Fig. 3). Thus, ZOEO inhibits the nutrient supply and damages the secondary metabolite production pathway of the *A. flavus* cells.

The anti-AFB_1_ efficacy of the ZOEO was reported in many reports but the detailed molecular mechanism was still lacking in the literature^48^. Therefore, to assess the comprehensive molecular mechanism of ZOEO, computational approach has been set out targeting the major gene products of the biosynthesis pathway. Since, the bioactivities of essential oils are related to their chemotype i.e., their major compound, hence all the computational studies were performed with the major compound (verbenol) for untangling the site of action of AFB_1_ biosynthesis in comparison with the commercial antifungal drug, amphotericin B. This is the first attempt to assess the multi-regime anti-AFB_1_ mechanism of verbenol targeting the *aflD, aflK*, and *aflP* gene products. Also, the safety limit profile of verbenol was explored using its ADMET profile (Table 1). The ADMET results revealed its favorable safety limit profile, biodegradable and non-toxic nature. The conclusions drawn were based on the comparative MD simulation approach of the target proteins in their ligand-bound and unbound forms.

**Figure 6:**
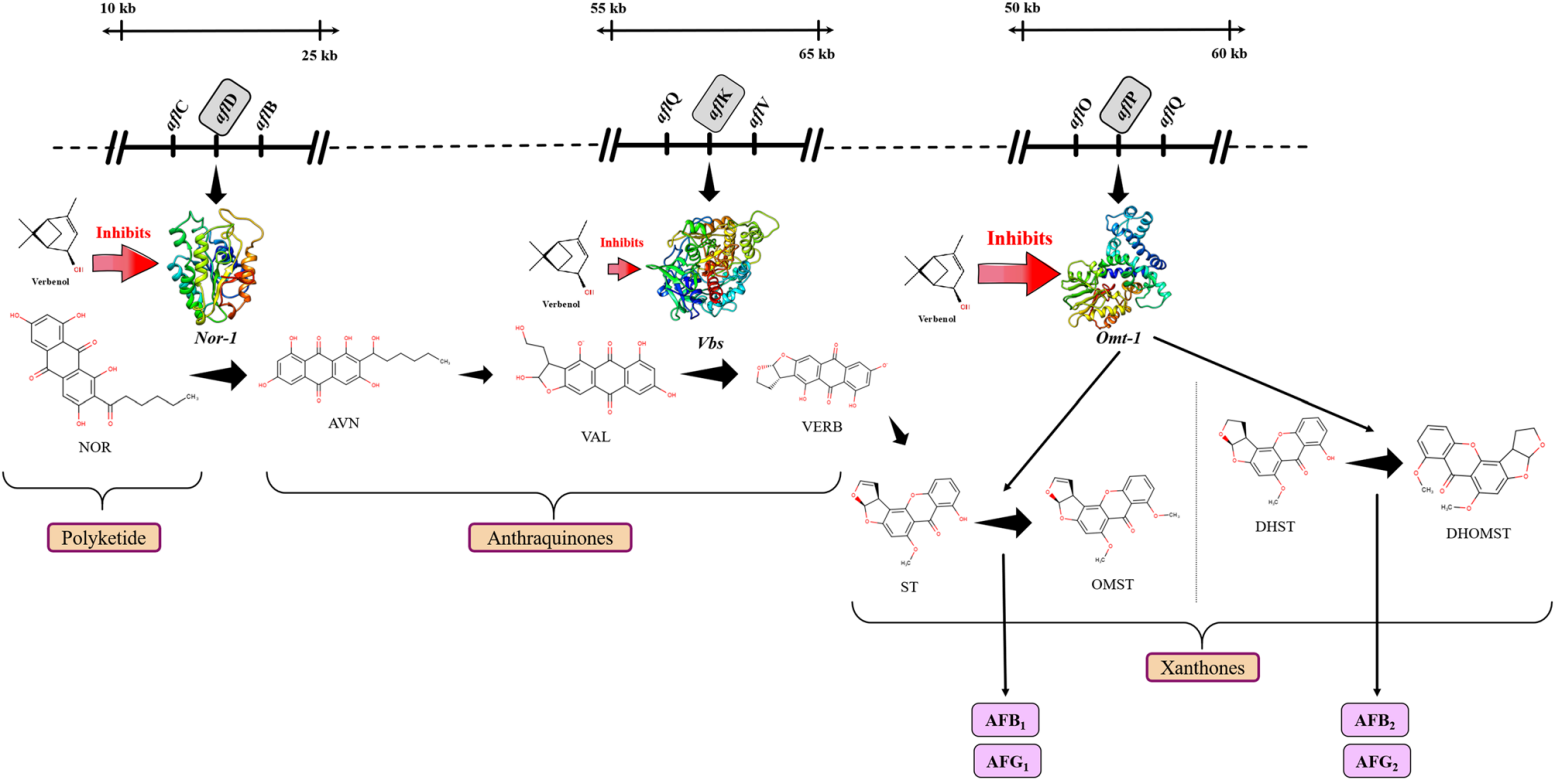
Schematic representation of molecular mechanism of action of anti-aflatoxigenic activity of verbenol.

The docking analysis confirmed the inhibitory binding of verbenol to all the target proteins. In absence of experimental data regarding the receptor-ligand complexes of target proteins, docking results were verified using the all-atom MD simulation approach^37^. It provides the ultimate details concerning the conformational changes of the target proteins upon ligand binding. These structural details of the docking-based receptor-ligand complexes of the target proteins led us to understand the molecular attributes of the verbenol-inhibitory action. RMSD and B-factor values confirmed the gain of stability to all the target proteins upon ligand binding (Fig. 4a). While, RMSF and Rg analysis showed the gain of compactness and less fluctuant motion only for the *Nor-1* and *Omt-1* on ligand binding, which is further verified by the higher number of the H-bonding in their ligand-bound state (Fig. 5a). In *Vbs*, there were fewer H-bonds in the ligand-bound state in comparison to its ligand unbound state, which might be the possible explanation of its relaxed gyradius and fluctuant motion of the protein residues. The H-bonds pattern between the verbenol and target proteins were also aligned with the above total H-bond results. These phenomena strike out the importance of the H-bonding for the stability of the protein 3D conformation. Essential dynamics analysis also provided some insight into the dynamical behavior of *Nor-1, Omt-1,* and *Vbs* in their ligand-bound and unbound form. The mode of significant motions occurring in the target proteins were captured via PC1 and PC2, which reveals a significant difference in major modes of motion in all three-target protein (*Nor-1, Omt-1,* and *Vbs*) in their ligand-bound form as compared to that of their ligand unbound form (Fig. 5b). These dynamics analysis was found to be consistent with the other analysis exploring the structural behaviors of the same. The MM/PBSA energy profiling preferred the *Nor-1*/verbenol and *Omt-1*/verbenol systems simultaneously as the better inhibitory action over the *Vbs*/verbenol system (Table 4). The Δ*G*_bind_ calculated with Poison Boltzmann (PB) model have the scheme of *Omt-1*/verbenol ~ *Nor-1*/verbenol > *Vbs*/verbenol. The other factors of thermo dynamical behavior viz. ΔE_MM_, solvation energy (ΔG_PBSA_), polar and non-polar contributions demonstrate a similar aspect. All of the above MD simulations results were converging at the inference that, in *Nor-1* and *Omt-1*, verbenol was bind at the interior of the protein structure while in *Vbs*, verbenol has the surface binding. Therefore, verbenol has the inhibitory action over the *Nor-1* and *Omt-1,* making them more stable than their natural form. While in *Vbs*, verbenol was also making the stability actions. However, its surface binding was causing the formation of the lower number of H-bonds that, in turn, perturbing the RMSF and gyradius data of the protein. The binding positions of receptor-ligand complexes obtained after MD calculations were shown in Fig. 4a. At last, the CaverDock analysis predicted the possible route of ligand placement into the active site of target proteins. The energy data obtained from the CaverDock reveals that the inhibitory action of the verbenol was only because of its binding at the active site rather than any tunnel blockage in the target proteins (Table 5). The predicted molecular mechanism of action of verbenol has been presented (Fig. 6).

## Conclusion

The findings of present investigation unravel the antifungal mode of action and molecular site of action of the verbenol-chemotype ZOEO against the biosynthesis of aflatoxin B_1_ using the biochemical and computational approaches. The investigation provided the antifungal and AFB_1_ inhibitory mode of action of ZOEO related with the damaging effects on the biochemical aspects (ergosterol production, MMP and carbohydrate catabolism) of the *A. flavus*. Further, the binding positions of verbenol with the target gene products (*Nor-1, Omt-1*, and *Vbs*) involved in aflatoxin B_1_ biosynthesis were investigated using docking procedures and validated with all-atom MD simulation approach. ADMET profile of verbenol revealed its favorable safety limit profile, biodegradable and non-toxic nature, strengthen its use as an eco-friendly antifungal preservative. In view of considerable antifungal, AFB_1_ inhibition and multi-regime inhibition efficiency of ZOEO and its compound verbenol, further practical investigations are warrant against *A*. *flavus* and AFB_1_ contamination in the food system.

## Methods

### Chemicals and equipment

All the chemicals used in the present study were procured from the Hi-Media Laboratories Pty Ltd., Mumbai, and SRL, Mumbai, India.

### Extraction and chemical profiling of ZOEO by GCMS

The rhizomes of *Zingiber officinale* were procured from the Botanical garden of the Banaras Hindu University, Uttar Pradesh state, India and authenticated using the relevant taxonomic literature^53^. A voucher specimen (Zin./Zin-149/2018) has been deposited in the herbarium of Department of Botany, Banaras Hindu University, Varanasi. The rhizomes (500 g) underwent hydro-distillation at 95 °C for 4 h using the Clevenger’s apparatus (Merck Specialties Pvt. Ltd., Mumbai, India), and the essential oil was collected in sterilized glass vial. The ZOEO was dried overnight using the anhydrous sodium sulfate (Na_2_SO_4_) and stored at 4 °C for chemical profiling^54^. The chemical profile of ZOEO was authenticated using the GC/MS (PerkinElmer, Turbomass Gold, USA) with the PerkinElmer Elite-5 column. The experimental procedure was similar to our previous published paper^4^.

### Efficacy against Aspergillus flavus and AFB_1_ contamination

The PN-05 strain of *Aspergillus flavus,* previously isolated in our laboratory, was chosen for the present investigation^52^. The antifungal activity of the ZOEO was tested against the *A. flavus* PN-05 using the poison food technique where the potato dextrose agar (PDA) plates were poisoned in a gradient manner from 0.1 to 0.6 μl/ml of ZOEO dissolved in 0.5 ml tween 20 (5%). Afterward, these plates were seeded with a 5 mm disc of the fungal hyphae at the center of each plate and incubated at 27 ± 2 °C for 7 days. The lowest ZOEO concentration having no visible fungal growth, was recorded as the minimum inhibitory concentration (MIC)^4^.

For investigating the anti-AFB_1_ efficacy, gradient concentrations of ZOEO (0.1 to 0.6 μl/ml) were separately mixed with the 24.5 ml of SMKY liquid medium (sucrose, 20 g; magnesium sulfate, 0.5 g; potassium nitrate, 3 g; yeast extract, 7 g; and distilled water, 1000 mL) and inoculated with a hundred microliter spore suspension of *A. flavus* PN-05. After the incubation of 10 ten days, the filtered media was used for the estimation of AFB_1_ content following the protocol of Prakash et al.^55^, based on the following formula:

$${\text{AFB}}_{1} {\text{ content }}\left( {{\upmu {\text{g}}}/{\text{ml}}} \right) = \frac{{{\text{D}} \times {\text{M}}}}{{{\text{E}} \times {\text{L }}}} \times 1000$$

where, D, absorbance; M, the molecular weight of AFB_1_ (312); E, the molar extinction coefficient of AFB1 (21,800); L, path length (1 cm cell was used).

### Antifungal mode of action

#### Effect of ZOEO on ergosterol production in the plasma membrane of *A. flavus*

The effect of different doses of ZOEO on the ergosterol production in the plasma membrane of the *A. flavus* was analyzed following the protocol of Kumar et al.^4^. The *A. flavus* PN-05 mycelia were treated with different dosses of ZOEO (0.125, 0.25 and 0.5 µl/ml) in SMKY medium and kept for five days’ incubation. Afterwards, mycelia harvest was done followed by addition of 5 ml of freshly prepared 25% alcoholic KOH solution and vortex for 2 min. Then after, the solutions were evaporated on water both at 85 °C for 4 h. followed by addition of 5 ml n-heptane and 2 ml sterile distilled water and vortex for 2 min. At last, optical density of the n-heptane layer was analyzed at 230 and 300 nm and ergosterol content was calculated using the following equation:

$$\% {\text{Ergosterol}} + \% 24\left( {28} \right){\text{ dehydroergosterol}} = \frac{{{\text{A}}_{282} /290}}{{\text{Pellet Weight}}},$$

$$\% 24\left( {28} \right){\text{ dehydroergosterol}} = \frac{{{\text{A}}_{230} /518}}{{\text{Pellet Weight}}},$$

$$\% {\text{Ergosterol}} = \left( {\frac{{{\text{A}}_{282} /290}}{{\text{Pellet Weight}}}} \right) - { }\left( {\frac{{{\text{A}}_{230} /518}}{{\text{Pellet Weight}}}} \right)$$

#### Effect of ZOEO on cellular ions

The 5-days incubated mycelia of *A. flavus* PN-05 was mixed with 20 ml of 0.85% saline solution followed by treatment of different doses of ZOEO (0.2, 0.4 and 0.6 µl/ml) and incubation at room temperature for 12 h. Afterwards, solution was filtered using the Whatman filter and filtrate was analyzed using atomic absorption spectrometry^56^.

#### Effect of ZOEO on mitochondrial membrane potential (MMP)

For analyzing the alterations in the membrane potential of mitochondria, the spore suspension (10^6^ spores/ml in tween 20 (0.5%)) of *A. flavus* PN-05 was treated with ZOEO at concentration of 0.2, 0.4 and 0.6 µl/ml for ten hours. Afterwards, samples were centrifuged at 5000 rpm for 5 min. and pellets were suspended in phosphate buffer saline solution. Subsequently, the staining of sample solutions was done using rhodamine 123 (1 μg/ml) for 15 min in the dark. Again, samples were centrifuged and mixed with the PBS solution followed by optical density quantification of Rh123 dye at 488 nm (excitation) and 525 nm (emission)^51^. A control set (without treatment) was also kept for the simultaneous assessment.

#### Effect of ZOEO on carbon-source utilization

The carbon-source utilization pattern of *A. flavus* PN-05 exposed to ZOEO (0.6 µl/ml) was assessed following the method of our previous publications^4^ with Biolog FF Microplate (94545, Hayward, CA).

### Molecular docking: the binding affinity of the receptor-ligand systems

#### Homology modeling and its authentication

To assess the molecular details of the inhibitory action of the ZOEO, computational analysis has been set out. Firstly, three-dimensional (3D) structures of the target proteins were required. Therefore, in lack of crystal structures of receptor-ligand complexes for target proteins, homology modeling technique was used to generate the 3D models of the target proteins. Firstly, the amino acid sequences of the target proteins were retrived from the NCBI database corresponding to the accession number EED51173 (*Nor-1*), EED51156 (*Omt-1*), and EED51154 (*Vbs*) in FASTA format. Further, the sequences were submitted to the PSI-BLAST and pGEN THREADER (http://bioinf.cs.ucl.ac.uk/psipred) web services for the template selection. Afterward, SWISS-MODEL web atmosphere (https://swissmodel.expasy.org)^20^ was used to generate the 3D models in reference to the selected templates. Validation and determining the acceptability of the generated 3D models were done with the Structure Analysis and Verification Server (SAVES) v5.0 at https://servicesn.mbi.ucla.edu/SAVES and ProTSAV function of SCFBIO^23^.

#### Binding site prediction

The active site prediction requires crystal structure of receptor–ligand complex. Hence in the absence crystal structure, computational analysis was deployed to predict the active site of the target proteins. It is the hydrophobic cavities (interior voids and surface pockets) of the proteins that are held responsible for their specific binding events^30^. Therefore, the theoretical determination of these cavities was necessary for predicting more accurate binding results. The Computed Atlas of Surface Topography of proteins (CASTp) 3.0 web facility was used to predict these kinds of active sites in the target proteins^27^. The modeled 3D structures of the proteins were submitted to the CASTp web facility to predict the necessary amino acids.

#### Ligand preparation and its ADMET profiling

The native structure of ligands (verbenol and amphotericin B) were retrieved from the PubChem database in .sdf format and processed to convert in the .pdbqt form for further computational analysis. In addition, Swiss Target Prediction^34^, Molinspirtation server, PREADMET (https://preadmet.bmdrc.kr), QikProp module (QikProp, Schrodinger LLC, NY, 2017), TEST (Toxicity Estimation Software Tool) of US Environmental Protection Agency and VEGA v1.1.5 (https://www.vegahub.eu) were assessed for the ADMET profiling and predicting the oral rat LD_50_, mutagenicity, drug-induced liver injury (hepatotoxicity), tumorigenicity, carcinogenicity, biodegradation, and other ecological risk assessment factors associated with verbenol.

#### Docking algorithm

The docking algorithm was performed using the AutoDock 4.2 docking protocols to determine the preferred interactive orientation of verbenol to the target gene products^57^. The docking parameters used were according to the standard genetic algorithm (GA) characters i.e., 10 runs of GA, population size 150, maximum number of ratings 250,000, maximum number of generations 27,000, and crossover rate 0.8. The docking environment were adjusted on the target proteins with the grid box having dimensions tabulated in Table 2. The grid-box dimensions were constructed for each target protein following their CASTp structure information. AutoDock 4.2 scoring function generates 120,000 ligand poses and rank ten superior conformations according to their binding energies. The selection of the receptor-ligand complexes was based on their binding energies and their positioning in the predictive active sites (visual inspection).

### MD simulation approach: validatory investigation of receptor–ligand complexes

#### Molecular dynamics simulation

*Nor-1*, *Omt-1,* and *Vbs* were subjected to all-atom MD simulation with and without ligand. So, a total of six systems were simulated. For MD simulation, all the initial structures were prepared in tleap module of Amber16 by utilizing the ff14SB force field^58^. The ligand forcefield parameters were generated using antechamber and gaff while the charges were generated using AM1-bcc method. TIP3P water model was used to solvate the simulation box with a padding distance of 12 Å and NaCl were used as counter ions to neutralizing the total charge of the system^59,60^. Joung and Cheatham ion parameters were used for Na+ and Cl− ions^61^. All MD simulations were performed using the Amber16 package^62^. For the electrostatic interaction calculation, Particle-mesh Ewald (PME) method was used. To restrain the bonds involving hydrogen SHAKE algorithm was used. For temperature and pressure control, Langevin dynamics and Berendson barostat were utilized^59^.

First, to minimize the energy of the six systems, a restrain was kept of protein–ligand complex, and only the solvent was minimized. After this round of minimization, the restrain potential was removed from complex, and the entire system was minimized. Followed by minimization, the gradual heating of the systems was performed at NVT with a 100 kcal restrain potential on complex and temperature was increased to 300 K in 300 ps. After heating, each system was subjected to equilibration for 1.8 ns in six cycles of 300 ps in which the restrain potential was subsequently reduced from 100 kcal/mod to 0 kcal/mol (100, 50, 20, 5, 0.5, 0 kcal/mol) at NPT^59^. After equilibration, all six systems were subjected to a production run of 300 ns each (except the *Nor-1*-ligand system which was simulated for 500 ns) at the NPT ensemble. A total of 2 µs productions simulation was performed, including all six systems under study.

#### Binding free energy calculation

The binding free energy calculation was done using each frame of last 100 ns of the trajectory of each system (target proteins with and without ligands) where no major deviations in RMSD at Cα atoms, its residue wise root mean square fluctuation, radius of gyration, and total number of intra-protein hydrogen bonds were observed. The calculations were performed using MMPBSA.py program of AmberTools^63^ with one-average MMPBSA method. The one average approach of MM/PBSA requires only one trajectory of the receptor-ligand complex and the ensemble average of both receptor and ligand is generated by removing appropriate atoms from the complex^64^. The binding free energy calculations were performed at internal dielectric constant of 2.0, external dielectric constant of 80.0 and ionic strength of 0.150 M. Entropy factor was not taken into consideration while calculation and other parameters like fill ratio (default value: 4) and scale (default value: 2) were used in default settings were used with default values.

#### Essential MD

Principle component analysis (PCA) was performed for *Nor-1*, *Omt-1*, and *Vbs* systems with and without verbenol. All the frames of trajectories were aligned around the protein backbone to remove the translational and rotational motion. Following this, the covariance matrix was generated, and eigenvalues and eigenvectors were generated by diagonalizing this matrix. After this, principal component 1 (PC1) and principal component 2 (PC2) were subsequently analyzed. All the calculation regarding PCA was done with CPPTRAJ module of amber16.

#### Protein-tunnel analysis

CaverDock tool (https://loschmidt.chemi.muni.cz/caverdock) has been assessed for the detection of the possible routes to the binding site within the protein and decipher the easiness in the ligand placement through these tunnels. Protein 3D models were used as the INPUT structures, and data obtained from active site analysis were used as the starting point for tunnel detection. Tunnels with a lower bottleneck radius, higher throughput, and lower E_Max_ and E_a_ values are selected for each target protein^44^.

## Supplementary Information

Supplementary Information.
