# Supplementary material for: Untangling the multi-regime molecular mechanism of verbenol-chemotype Zingiber officinale essential oil against Aspergillus flavus and aflatoxin B1
Source: Sci Rep. 2021 Mar 25;11:6832. doi: 10.1038/s41598-021-86253-8 (PMC7994644; doi:10.1038/s41598-021-86253-8)
Supplement: Supplementary file 1 — Supplementary Information. [file 41598_2021_86253_MOESM1_ESM.docx]

**Title Page**

**Untangling the multi-regime molecular mechanism of verbenol-chemotype *Zingiber officinale* essential oil against *Aspergillus flavus* and aflatoxin B_1_**

**Authors: Prem Pratap Singh^a^, Atul Kumar Jaiswal^b^, Akshay Kumar^a^, Vishal Gupta^a^, and Bhanu Prakash^a^***

**^a^Centre of Advanced Study in Botany, Institute of Science, Banaras Hindu University, Varanasi, 221005, India.**

**^b^Department of Biochemical Engineering and Biotechnology, Indian Institute of Technology Delhi, New Delhi, 110016, India.**

***Correspondence**

**E-mail addresses:** [bprakash@bhu.ac.in](mailto:bprakash@bhu.ac.in); [bhanubhu08@gmail.com](mailto:bhanubhu08@gmail.com)

**Telephone:** +91-9794113055; +91-9482016540

**
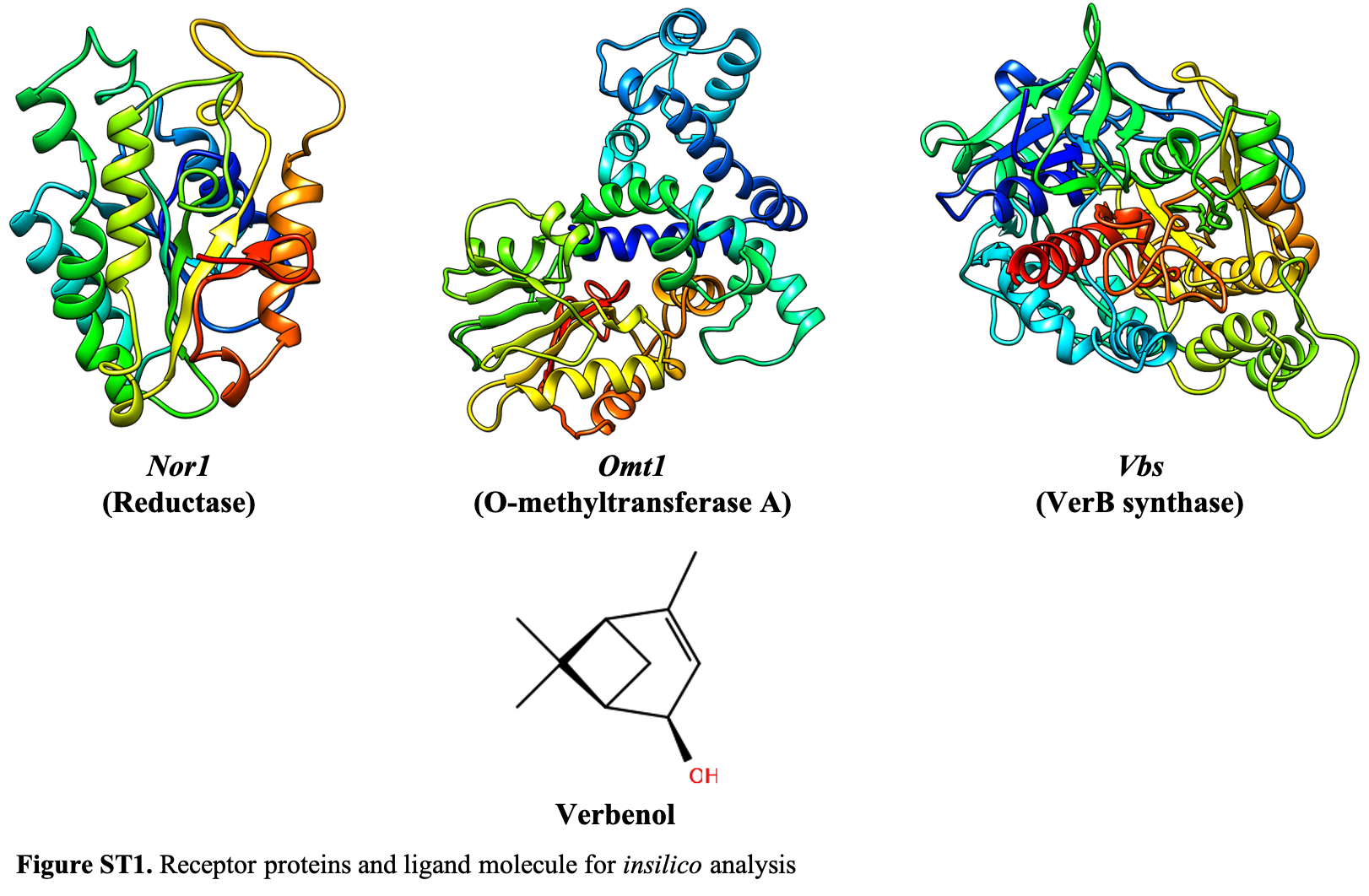
**


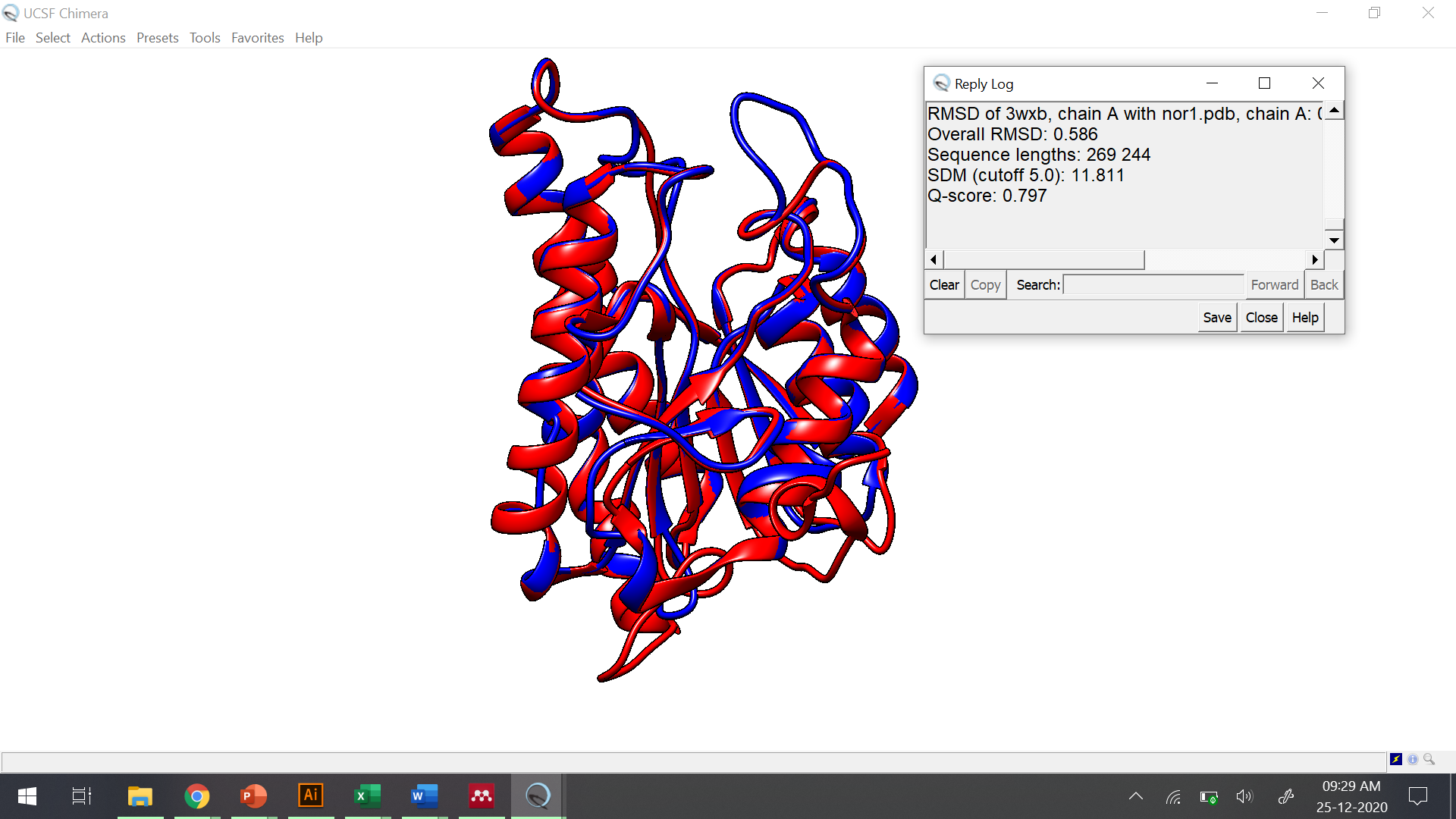


1. ***Nor-1* superimposed with protein PDB ID: 3WXB**


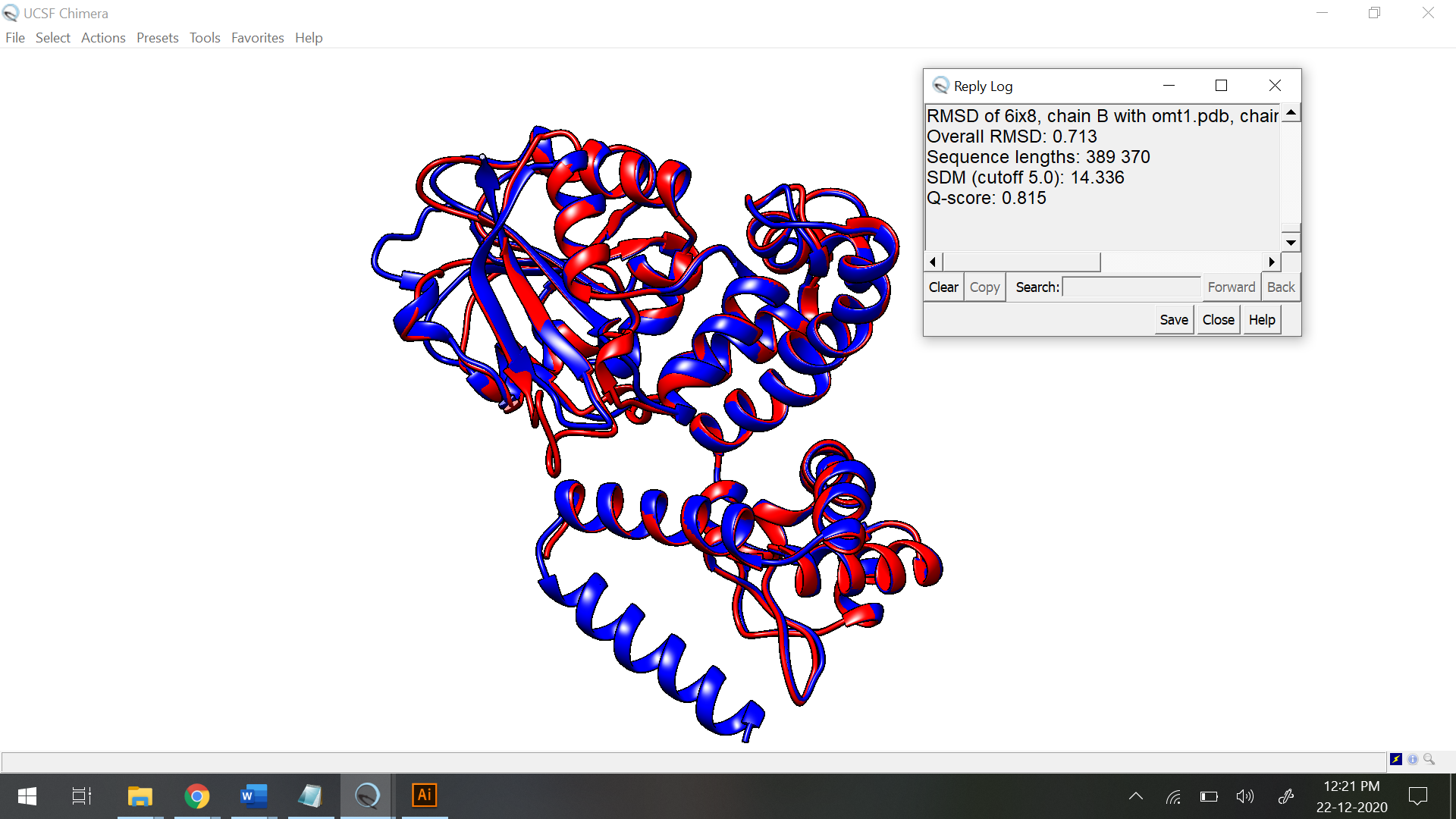


1. ***Omt-1* superimposed with protein PDB ID: 6IX8**


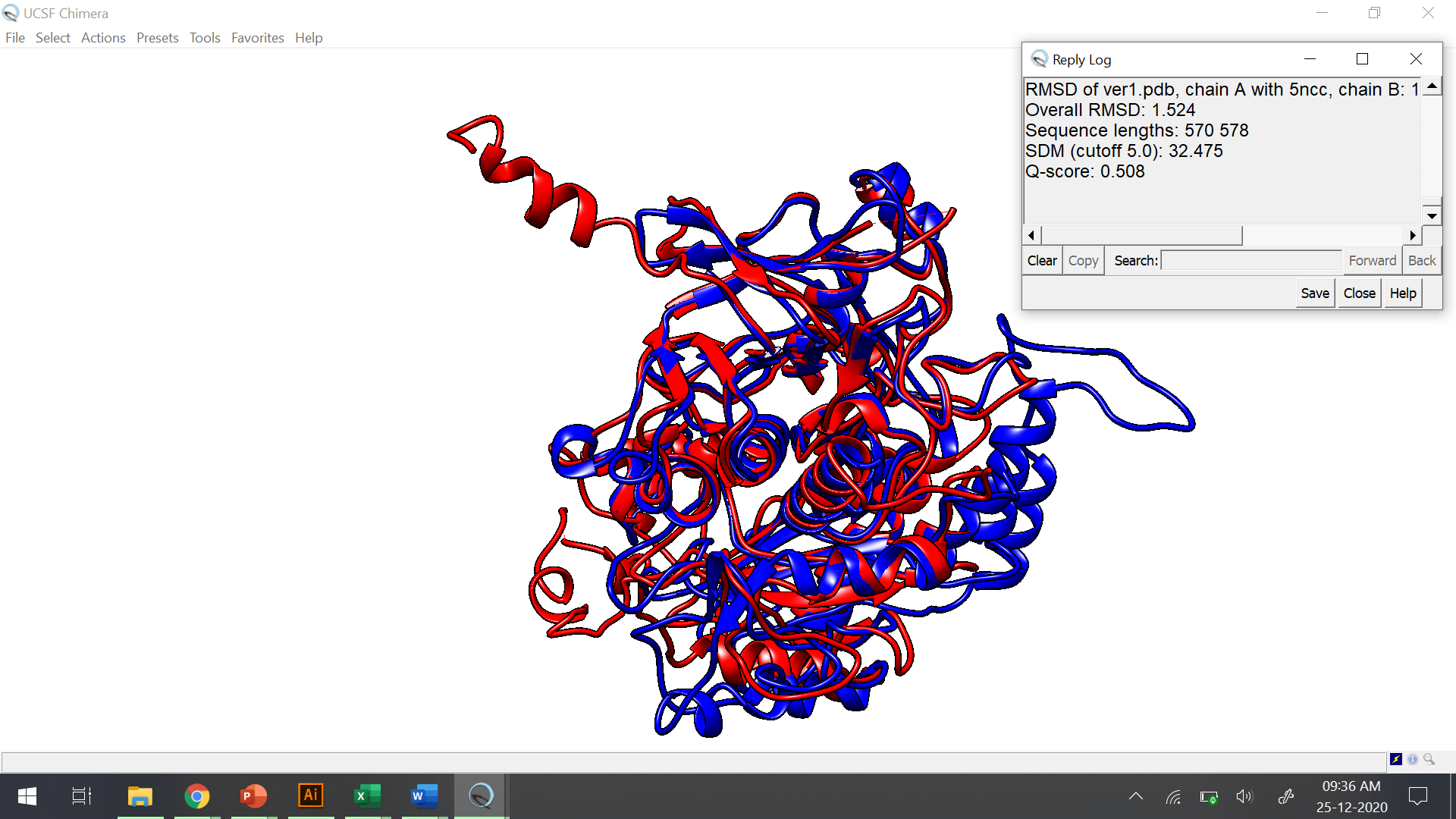


1. ***Vbs* superimposed with protein PDB ID: 5NCC**

**Figure ST2. Modelled structure of target proteins, superimposed with their template crystal stuctures**

**
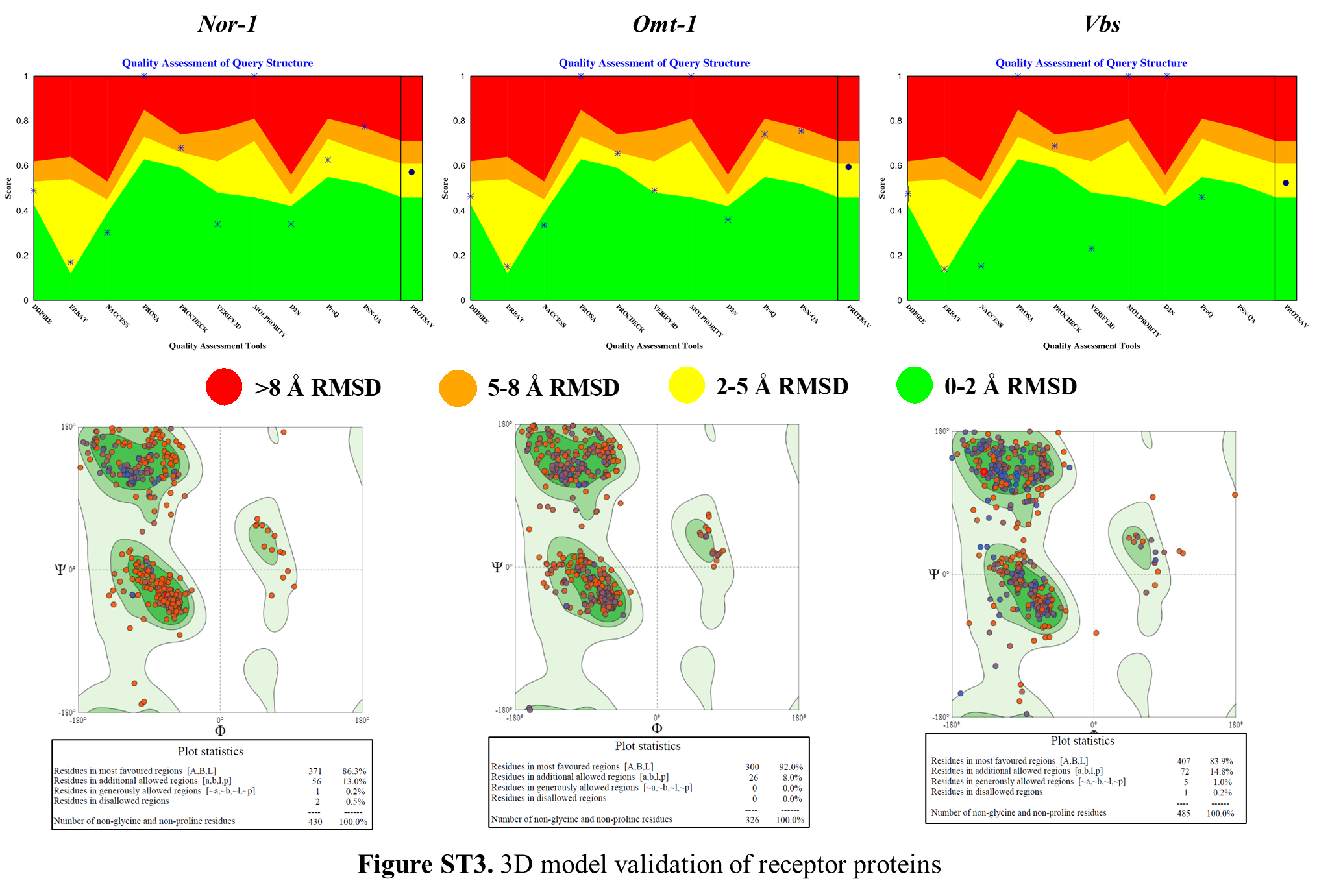
**

**
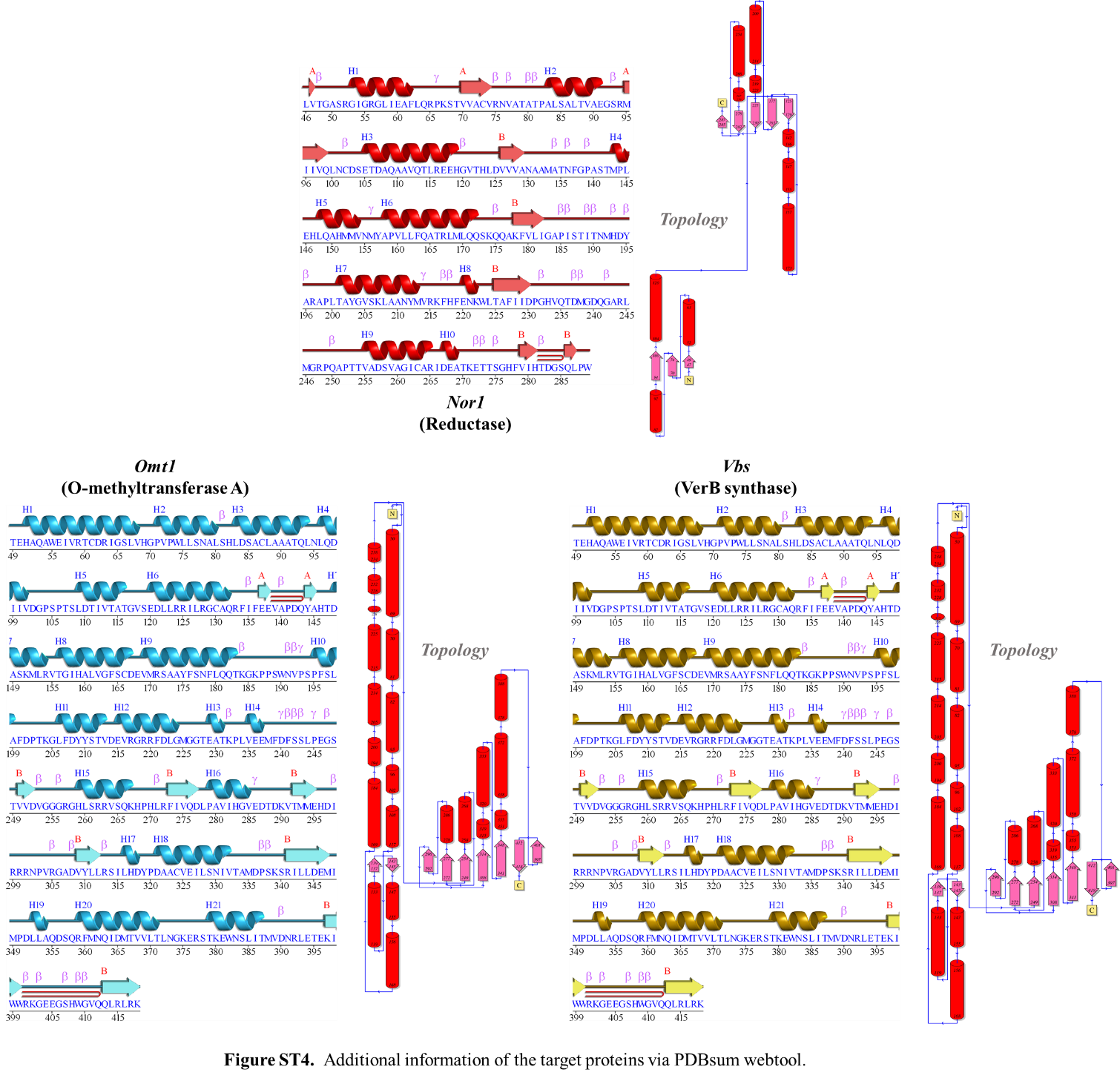
**

**
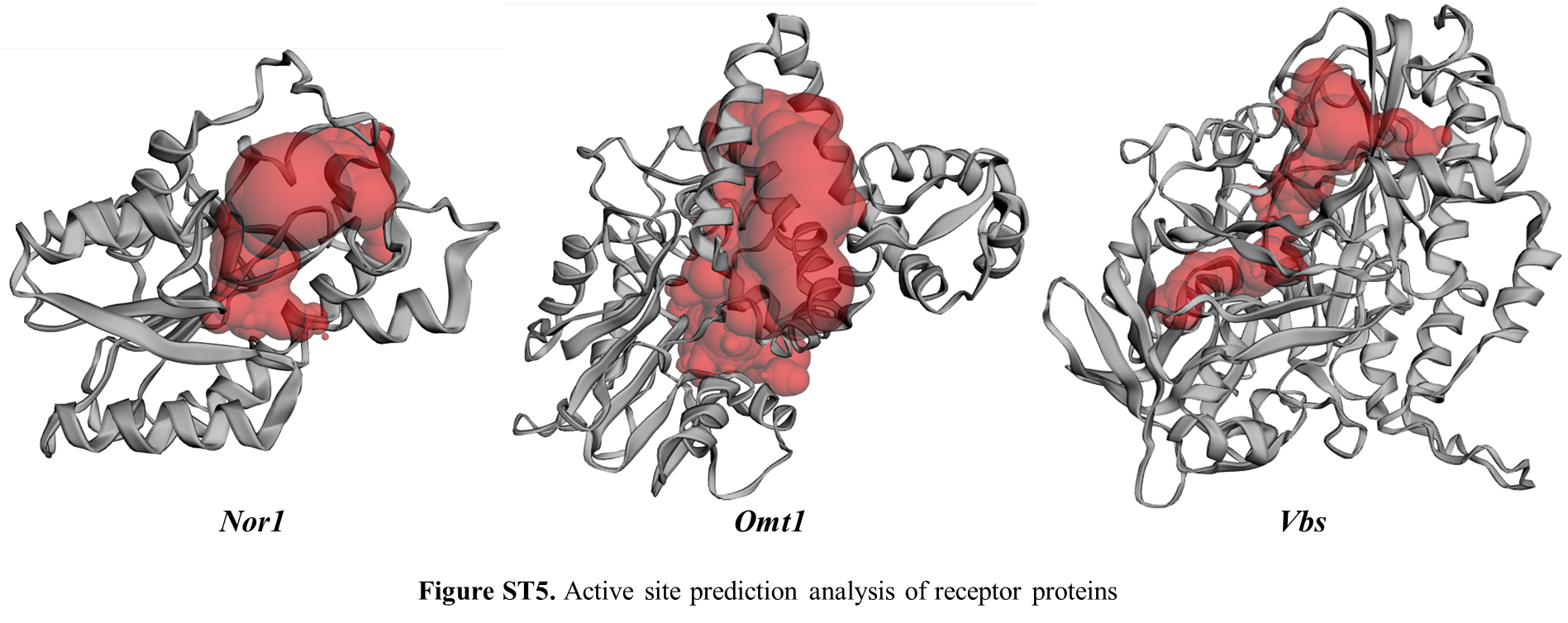
**

**
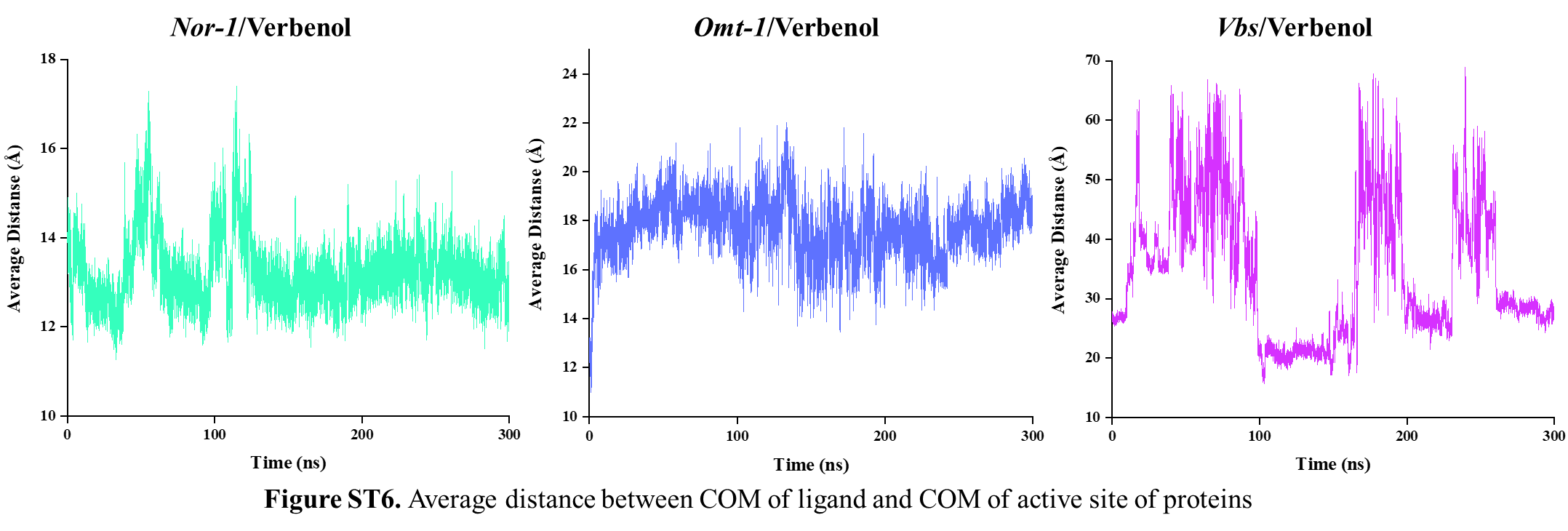
**

**Table ST1a**. Ligand-protein H-bond details for *Nor-1*/verbenol system

| Donor | Acceptor | Occupancy |
| --- | --- | --- |
| ILE9-Main-N | LIG245-Main-O | 0.31% |
| GLY10-Main-N | LIG245-Main-O | 0.76% |
| LIG245-Main-O | ASN85-Side-OD1 | 11.46% |
| ASN85-Side-ND2 | LIG245-Main-O | 2.51% |
| LIG245-Main-O | GLY4-Main-O | 4.42% |
| LIG245-Main-O | ARG7-Main-O | 0.46% |
| SER6-Main-N | LIG245-Main-O | 0.00% |
| ARG7-Main-N | LIG245-Main-O | 0.02% |
| ALA87-Main-N | LIG245-Main-O | 2.40% |
| LIG245-Main-O | SER6-Side-OG | 0.01% |
| LIG245-Main-O | ASN85-Main-O | 20.60% |
| LIG245-Main-O | ALA89-Main-O | 2.32% |
| ASN85-Side-ND2 | LIG245-Side-C3 | 0.01% |
| LIG245-Main-O | ASP192-Side-OD2 | 0.22% |
| ASP192-Main-N | LIG245-Main-O | 0.11% |
| LIG245-Main-O | ASP192-Side-OD1 | 0.57% |
| MET88-Main-N | LIG245-Main-O | 0.70% |
| LIG245-Main-O | THR90-Main-O | 0.02% |
| PHE92-Main-N | LIG245-Main-O | 0.00% |
| LIG245-Main-O | ASN91-Main-O | 0.00% |
| LIG245-Main-O | MET88-Main-O | 0.05% |
| LIG245-Main-O | TYR158-Side-OH | 0.12% |
| TYR158-Side-OH | LIG245-Main-O | 2.81% |
| TYR158-Side-OH | LIG245-Side-C2 | 0.01% |
| LIG245-Main-O | GLY138-Main-O | 0.93% |
| LIG245-Main-O | THR191-Side-OG1 | 0.10% |
| LIG245-Main-O | ILE137-Main-O | 0.11% |
| LYS162-Side-NZ | LIG245-Main-O | 0.05% |
| LIG245-Main-O | ASN85-Side-ND2 | 0.00% |
| THR191-Side-OG1 | LIG245-Main-O | 0.29% |
| LIG245-Main-O | MET88-Side-SD | 0.01% |

**Table ST1b**. Ligand-protein H-bond details for *Omt-1*/verbenol system

| Donor | Acceptor | Occupancy |
| --- | --- | --- |
| ARG209-Main-N | LIG371-Main-O | 0.23% |
| LIG371-Main-O | HIE211-Side-ND1 | 0.03% |
| LIG371-Main-O | GLY207-Main-O | 0.02% |
| LIG371-Main-O | PHE173-Main-O | 0.03% |
| LIG371-Main-O | SER163-Side-OG | 7.69% |
| ARG209-Side-NH2 | LIG371-Main-O | 0.03% |
| ARG209-Side-NH1 | LIG371-Main-O | 10.19% |
| LIG371-Main-O | ASP174-Side-OD2 | 2.31% |
| LIG371-Main-O | ASP174-Side-OD1 | 3.48% |
| LIG371-Main-O | GLY170-Main-O | 0.87% |
| LIG371-Main-O | ASP160-Side-OD1 | 1.54% |
| LIG371-Main-O | ALA232-Main-O | 1.02% |
| LIG371-Main-O | ASP174-Side-CG | 0.16% |
| SER163-Side-OG | LIG371-Main-O | 0.57% |
| LIG371-Main-O | ASP160-Side-CG | 0.02% |
| ARG209-Side-NE | LIG371-Main-O | 0.49% |
| LIG371-Main-O | ASP160-Side-OD2 | 0.74% |
| ARG171-Side-NH2 | LIG371-Main-O | 0.22% |
| LIG371-Main-O | MET177-Side-SD | 0.01% |
| LIG371-Main-O | VAL233-Main-O | 1.12% |
| LIG371-Main-O | HIE235-Main-O | 0.01% |
| SER163-Side-OG | LIG371-Side-C3 | 0.01% |
| HIE235-Side-NE2 | LIG371-Main-O | 0.91% |
| LIG371-Main-O | HIE235-Side-ND1 | 0.18% |
| HIE235-Side-NE2 | LIG371-Side-C2 | 0.01% |
| LIG371-Main-O | PHE159-Main-O | 0.01% |
| ARG171-Side-NH1 | LIG371-Main-O | 0.22% |
| HIE235-Main-N | LIG371-Main-O | 0.00% |
| LIG371-Main-O | SER163-Side-CB | 0.07% |
| LIG371-Main-O | HIE235-Side-NE2 | 0.00% |
| LIG371-Main-O | GLY236-Main-O | 0.06% |
| LIG371-Main-O | GLU238-Side-OE2 | 0.67% |
| LIG371-Main-O | GLU238-Side-OE1 | 0.67% |
| LIG371-Main-O | GLU238-Side-CD | 0.02% |
| GLY178-Main-N | LIG371-Main-O | 0.00% |
| ARG209-Side-NH1 | LIG371-Side-C2 | 0.02% |
| GLY236-Main-N | LIG371-Main-O | 0.00% |

**Table ST1c**. Ligand-protein H-bond details for *Vbs*/verbenol system

| Donor | Acceptor | Occupancy |
| --- | --- | --- |
| LIG571-Main-O | TYR72-Main-O | 2.69% |
| LIG571-Main-O | PRO441-Main-O | 0.01% |
| VAL443-Main-N | LIG571-Main-O | 0.20% |
| LIG571-Main-O | ASP444-Side-OD2 | 0.00% |
| GLN301-Side-NE2 | LIG571-Main-O | 0.03% |
| LIG571-Main-O | HIE298-Side-ND1 | 0.02% |
| LIG571-Main-O | GLN301-Side-OE1 | 0.04% |
| LIG571-Main-O | ASN433-Side-OD1 | 0.05% |
| LIG571-Main-O | GLN301-Main-O | 0.09% |
| LIG571-Main-O | ASN433-Main-O | 0.09% |
| LIG571-Main-O | GLU300-Main-O | 0.01% |
| LIG571-Main-O | GLU300-Side-OE1 | 0.00% |
| LIG571-Main-O | GLY303-Main-O | 0.26% |
| LIG571-Main-O | VAL306-Main-O | 0.06% |
| ARG307-Side-NH2 | LIG571-Main-O | 0.00% |
| ARG307-Side-NH1 | LIG571-Main-O | 0.01% |
| LIG571-Main-O | ASP251-Side-OD1 | 0.00% |
| LIG571-Main-O | ASP251-Side-OD2 | 0.01% |
| LIG571-Main-O | ASN435-Side-OD1 | 0.08% |
| ARG246-Side-NH2 | LIG571-Main-O | 0.31% |
| ARG246-Side-NE | LIG571-Main-O | 0.04% |
| LIG571-Main-O | GLU266-Side-OE2 | 0.07% |
| LIG571-Main-O | GLU266-Side-OE1 | 0.33% |
| LYS245-Side-NZ | LIG571-Main-O | 0.12% |
| ASN435-Side-ND2 | LIG571-Main-O | 0.38% |
| LIG571-Main-O | VAL290-Main-O | 0.19% |
| LIG571-Main-O | ASN435-Main-O | 0.16% |
| ARG246-Side-NH1 | LIG571-Main-O | 0.03% |
| LIG571-Main-O | GLU266-Main-O | 0.02% |
| LIG571-Main-O | GLN268-Side-OE1 | 0.05% |
| GLN268-Side-NE2 | LIG571-Main-O | 0.01% |
| THR259-Side-OG1 | LIG571-Main-O | 0.01% |
| LIG571-Main-O | ASP436-Side-OD2 | 0.02% |
| LIG571-Main-O | ASP436-Side-CG | 0.00% |
| LIG571-Main-O | ASP436-Side-OD1 | 0.02% |
| LIG571-Main-O | GLU266-Side-CD | 0.00% |
| LIG571-Main-O | TRP267-Main-O | 0.01% |
| GLN1-Main-N | LIG571-Main-O | 0.01% |
| SER2-Main-N | LIG571-Main-O | 0.03% |
| LIG571-Main-O | ALA438-Main-O | 0.03% |
| LIG571-Main-O | GLY264-Main-O | 0.03% |
| LIG571-Main-O | ASP263-Main-O | 0.01% |
| LIG571-Main-O | GLY60-Main-O | 0.00% |
| LYS61-Side-NZ | LIG571-Main-O | 0.01% |
| GLY60-Main-N | LIG571-Main-O | 0.01% |
| LIG571-Main-O | ASP58-Side-OD1 | 0.00% |
| ARG241-Side-NH2 | LIG571-Main-O | 0.00% |
| LIG571-Main-O | ASP263-Side-OD2 | 0.02% |
| ASN64-Side-ND2 | LIG571-Main-O | 0.01% |
| LIG571-Main-O | ASN64-Side-OD1 | 0.00% |
| ARG241-Side-NH1 | LIG571-Main-O | 0.00% |
| LIG571-Main-O | ASP263-Side-OD1 | 0.00% |
| LIG571-Main-O | SER197-Main-O | 0.18% |
| LIG571-Main-O | ALA158-Main-O | 0.05% |
| ARG105-Side-NH1 | LIG571-Main-O | 0.24% |
| LIG571-Main-O | LEU129-Main-O | 0.01% |
| LIG571-Main-O | PHE159-Side-CE1 | 0.00% |
| LYS134-Side-NZ | LIG571-Main-O | 0.05% |
| LYS134-Side-CE | LIG571-Main-O | 0.00% |
| LIG571-Main-O | PHE159-Side-CE2 | 0.00% |
| GLN133-Side-NE2 | LIG571-Main-O | 0.03% |
| LIG571-Main-O | PHE159-Main-O | 0.01% |
| LIG571-Main-O | GLN133-Side-OE1 | 0.07% |
| ARG105-Side-NH2 | LIG571-Main-O | 0.05% |
| LIG571-Main-O | ASP126-Side-OD2 | 0.11% |
| LIG571-Main-O | ASP126-Main-O | 0.04% |
| LIG571-Main-O | GLN194-Side-OE1 | 0.02% |
| LIG571-Main-O | GLU192-Main-O | 0.01% |
| GLN194-Side-NE2 | LIG571-Main-O | 0.04% |
| GLY195-Main-N | LIG571-Main-O | 0.19% |
| LIG571-Main-O | SER197-Side-OG | 0.01% |
| SER197-Side-OG | LIG571-Main-O | 0.07% |
| LIG571-Main-O | THR151-Main-O | 0.24% |
| LIG571-Main-O | LEU153-Main-O | 0.02% |
| LIG571-Main-O | ALA150-Main-O | 0.09% |
| LIG571-Main-O | THR157-Side-OG1 | 0.01% |
| LIG571-Main-O | ALA152-Main-O | 0.03% |
| ASN198-Side-ND2 | LIG571-Main-O | 0.03% |
| TRP125-Side-NE1 | LIG571-Main-O | 0.02% |
| LIG571-Main-O | ASN198-Main-O | 0.00% |
| LIG571-Main-O | GLU332-Side-OE1 | 0.00% |
| LIG571-Main-O | ASN413-Side-OD1 | 0.15% |
| ASN413-Side-ND2 | LIG571-Main-O | 0.01% |
| LIG571-Main-O | TYR414-Side-OH | 0.01% |
| LIG571-Main-O | THR326-Main-O | 0.04% |
| TYR497-Side-OH | LIG571-Main-O | 0.04% |
| LIG571-Main-O | TYR497-Side-OH | 0.00% |
| LIG571-Main-O | ASP403-Main-O | 0.07% |
| ASN59-Side-ND2 | LIG571-Main-O | 0.03% |
| LIG571-Main-O | TYR414-Side-CE1 | 0.00% |
| LIG571-Main-O | THR326-Side-OG1 | 0.02% |
| THR326-Side-OG1 | LIG571-Main-O | 0.01% |
| LIG571-Main-O | ASN59-Side-OD1 | 0.01% |
| LIG571-Main-O | ASN54-Side-OD1 | 0.02% |
| ASN54-Side-ND2 | LIG571-Main-O | 0.05% |
| LIG571-Main-O | ASP58-Side-OD2 | 0.03% |
| LIG571-Main-O | TYR414-Side-CD1 | 0.00% |
| TYR414-Side-OH | LIG571-Main-O | 0.00% |
| LIG571-Main-O | GLU370-Main-O | 0.01% |
| HIE372-Main-N | LIG571-Main-O | 0.01% |
| LIG571-Main-O | GLU370-Side-OE1 | 0.03% |
| LIG571-Main-O | GLU370-Side-OE2 | 0.04% |
| LIG571-Main-O | LEU302-Main-O | 0.04% |
| ASN433-Side-ND2 | LIG571-Main-O | 0.00% |
| SER434-Side-OG | LIG571-Main-O | 0.02% |
| SER434-Side-OG | LIG571-Side-C2 | 0.00% |
| LIG571-Main-O | GLN354-Main-O | 0.02% |
| LIG571-Main-O | GLY357-Main-O | 0.01% |
| LIG571-Main-O | GLU353-Main-O | 0.05% |
| GLN354-Side-NE2 | LIG571-Main-O | 0.02% |
| LIG571-Main-O | GLU350-Side-OE2 | 0.01% |
| LIG571-Main-O | GLU353-Side-OE2 | 0.00% |
| LIG571-Main-O | GLN354-Side-OE1 | 0.04% |
| LIG571-Main-O | GLU455-Side-OE2 | 0.42% |
| LIG571-Main-O | GLU455-Side-OE1 | 0.22% |
| LIG571-Main-O | GLU455-Side-CD | 0.01% |
| LIG571-Main-O | ALA347-Main-O | 0.02% |
| LYS341-Side-NZ | LIG571-Main-O | 0.07% |
| ARG452-Side-NE | LIG571-Main-O | 1.59% |
| ARG452-Side-NH1 | LIG571-Main-O | 0.32% |
